# Supplementary material for: Optimization of individualized faricimab dosing for patients with diabetic macular edema: Protocol for the SWAN open-label, single-arm clinical trial
Source: PLoS One. 2024 Oct 10;19(10):e0311484. doi: 10.1371/journal.pone.0311484 (PMC11466402; doi:10.1371/journal.pone.0311484)
Supplement: S1 Table — BCVA, best-corrected visual acuity; CFP, color fundus photography; CST, central subfield thickness; DME, diabetic macular edema; IMP, investigational medicinal product; PDR, proliferative diabetic retinopathy; PRP, panretinal photocoagulation; SD-OCT, spectral-domain optical coherence tomography; VEGF, vascular endothelial growth factor; YAG, yttrium-aluminum-garnet. *Postmenarchal women who have not reached postmenopausal status (amenorrhea for at least 12 consecutive months with no cause other than menopause), and are not permanently infertile by surgery (removal of ovaries, fallopian tubes and/or uterus) or other causes as determined by the investigator or co-investigator (e.g., Müllerian duct dysplasia) considered women of childbearing potential. According to this provision, women with unilateral tubal ligation are considered women of childbearing potential. †Examples of contraceptive methods with annual failure rates of less than 1% include bilateral tubal ligation, male sterilization, hormonal contraceptives that inhibit ovulation, hormone-releasing intrauterine devices, and copper-added intrauterine devices. The reliability of sexual abstinence should be evaluated with respect to the duration of the clinical research and each patient’s preferences and normal lifestyle. Cyclic abstinence (calendar, ovulation day, symptomatic temperature, postovulation, etc.), and external ejaculation are not adequate contraceptive methods. ‡Systemic anti-VEGF therapy; systemic drugs known to cause macular edema (fingolimod, tamoxifen); intravitreal administration of anti-VEGF agents (other than faricimab) into study eye; intravitreal, periocular (subtenon), steroid implants, or chronic topical ocular corticosteroids into study eye; photodynamic therapy to study eye; micropulse, and focal or grid photocoagulation in the study eye; vitreous surgery or PRP in the study eye; kallidinogenase (for improvement of symptoms of circulatory disturbance of the retinal choroid); other expe [file pone.0311484.s001.pdf]

Supporting Information

Table S1. SWAN trial eligibility criteria.

**Inclusion criteria**	
General inclusion criteria	• Provide signed informed consent<br>• Age ≥18 years at the time of consent<br>• Confirmed diagnosis of diabetes mellitus (type 1 or type 2)<br>• Ability and willingness to undertake all scheduled visits and assessments
Ocular inclusion criteria for the study eye	• Macular thickening secondary to DME involving the center of the fovea with CST ≥325 μm, as measured on Spectralis SD-OCT, or ≥315 μm, as measured on Cirrus SD-OCT or Topcon SD-OCT (or other equivalent OCTs) at screening<br>• BCVA of 0.0625 ~ 0.7 (decimal visual acuity) on visual acuity test conducted at screening<br>• Sufficiently clear optic media and adequate pupillary dilatation to allow acquisition of good quality CFP and other imaging modalities
**Exclusion criteria**	
General exclusion criteria	• History of severe allergic reaction or anaphylactic reaction to a biologic agent or known hypersensitivity to faricimab and any of its excipients, mydriatic eye drops, anesthetics, or antimicrobials

	• History of other diseases, other nondiabetic metabolic dysfunction, physical examination finding, historical or current clinical laboratory finding giving reasonable suspicion of a condition that contraindicates the use of the faricimab or that might affect interpretation of the results of the trial or renders the patient at high risk for treatment complications in the opinion of the investigator or co-investigator<br>• Active cancer within the past 12 months except for appropriately treated carcinoma *in situ* of the cervix, nonmelanoma skin carcinoma, and prostate cancer with a Gleason score of ≤6 and stable prostate-specific antigen for >12 months<br>• Systemic treatment for suspected or active systemic infection<br>• Participation in an investigational study that involves treatment with any drug or device (except for vitamins and minerals) within 3 months before day 1<br>• Administration of systemic pro-angiogenic treatments for the peripheral or coronary ischemia (e.g., limb ischemia or myocardial infarction) within 3 months before day 1<br>• Unwilling to comply with the trial or follow-up procedures<br>• Renal failure requiring renal transplant, hemodialysis, or peritoneal dialysis, or anticipated to require hemodialysis or peritoneal dialysis at any time during the trial

	• Uncontrolled blood pressure (defined as systolic >180 mmHg and/or diastolic >100 mmHg while a patient is at rest). If a patient's initial reading exceeds these values, a second reading may be obtained later the same day or on another day during the screening period<br>• Stroke (cerebral vascular accident) or myocardial infarction within 6 months before day 1<br>• Pregnancy or breastfeeding, or intention to become pregnant during the trial<br>• Women of childbearing potential* who do not agree to remain abstinent (refrain from heterosexual intercourse) or use acceptable contraceptive methods that result in a failure rate of <1% per year[†] during the treatment period and for at least 38 days after the final dose of the IMP<br>• Requirement for continuous use of any prohibited concomitant medications or therapies[‡]<br>• Not receiving treatment for diabetes
Ocular exclusion criteria for the study eye	• High-risk PDR in the study eye (using any of the following established criteria for high-risk PDR):<br>– Any vitreous or preretinal hemorrhage<br>– Neovascularization elsewhere ≥1/2 disc area within an area equivalent to the mydriatic ETDRS 7 fields on clinical examination or CFPs<br>– Neovascularization at disc ≥1/3 disc area on clinical examination<br>• Tractional retinal detachment, preretinal fibrosis, vitreomacular traction syndrome, or epiretinal membrane involving the fovea or disrupting the macular architecture in the study eye

	• Active rubeosis<br>• Uncontrolled glaucoma<br>• History of retinal detachment or macular hole (Stage 3 or 4)<br>• Aphakia or implantation of anterior chamber intraocular lens<br>• Intravitreal administration of anti-VEGF agents within 3 months before day 1 (applicable to patients whose study eyes were previously treated with intravitreal anti-VEGF agents), or any intravitreal administration of anti-VEGF agents to study eye before day 1 (applicable for treatment-naïve patients). Enrollment of patients who have a medication history of intravitreal administration of anti-VEGF agents should be no more than 25% of the total<br>• History of PRP, macular laser (focal, grid, or micropulse), any cataract surgery or treatment for complications of cataract surgery with steroids or YAG laser capsulotomy within 3 months before day 1<br>• Any other intraocular surgery (e.g., corneal transplantation, glaucoma filtration, pars plana vitrectomy, corneal transplant, or radiotherapy)<br>• Any intravitreal or periocular (subtenon) corticosteroid treatment within 6 months before day 1<br>• Treatment for other retinal diseases that can lead to macular edema<br>• Prior intravitreal administration of faricimab

Ocular exclusion criteria for the nonstudy eye	• Nonfunctioning nonstudy eye, defined as either:<br>– BCVA of hand motion or worse<br>– No physical presence of nonstudy eye (i.e., monocular)
Ocular exclusion criteria for both eyes	• Any history of idiopathic or immune-mediated uveitis in either eye<br>• Active ocular inflammation or suspected or active ocular or periocular infection in either eye on day 1
Exclusion criteria for concurrent ocular conditions	• Any current or history of ocular disease other than DME that may confound the assessment of the macula or affect central vision in the study eye (choroidal neovascularization, age-related macular degeneration, retinal vein occlusion, uveitis, angioid streaks, histoplasmosis, active or inactive cytomegalovirus, pathological myopia, retinal detachment, retinal embolus, macular traction, macular hole, and other)<br>• Any current ocular condition that, in the opinion of the investigator or co-investigator, is currently causing or could be expected to contribute to irreversible vision loss because of a cause other than DME in the study eye (e.g., foveal atrophy, foveal fibrosis, pigment abnormalities, dense subfoveal hard exudates, or other nonretinal conditions)

BCVA, best-corrected visual acuity; CFP, color fundus photography; CST, central subfield thickness; DME, diabetic macular edema; IMP, investigational medicinal product; PDR, proliferative diabetic retinopathy; PRP, panretinal photocoagulation; SD-OCT, spectral-domain optical coherence tomography; VEGF, vascular endothelial growth factor; YAG, yttrium-aluminum-garnet.

*Postmenarchal women who have not reached postmenopausal status (amenorrhea for at least 12 consecutive months with no cause other than menopause), and are not permanently infertile by surgery (removal of ovaries, fallopian tubes and/or uterus) or other causes as determined by the investigator or co-investigator (e.g., Müllerian duct dysplasia) considered women of childbearing potential. According to this provision, women with unilateral tubal ligation are considered women of childbearing potential.

†Examples of contraceptive methods with annual failure rates of less than 1% include bilateral tubal ligation, male sterilization, hormonal contraceptives that inhibit ovulation, hormone-releasing intrauterine devices, and copper-added intrauterine devices. The reliability of sexual abstinence should be evaluated with respect to the duration of the clinical research and each patient's preferences and normal lifestyle. Cyclic abstinence (calendar, ovulation day, symptomatic temperature, postovulation, etc.), and external ejaculation are not adequate contraceptive methods.

‡Systemic anti-VEGF therapy; systemic drugs known to cause macular edema (fingolimod, tamoxifen); intravitreal administration of anti-VEGF agents (other than faricimab) into study eye; intravitreal, periocular (subtenon), steroid implants, or chronic topical ocular corticosteroids into study eye; photodynamic therapy to study eye; micropulse, and focal or grid photocoagulation in the study eye; vitreous surgery or PRP in the study eye; kallidinogenase (for improvement of symptoms of circulatory disturbance of the retinal choroid); other experimental therapies (except those comprising vitamins and minerals).
