# Supplementary material for: Optimization of individualized faricimab dosing for patients with diabetic macular edema: Protocol for the SWAN open-label, single-arm clinical trial
Source: PLoS One. 2024 Oct 10;19(10):e0311484. doi: 10.1371/journal.pone.0311484 (PMC11466402; doi:10.1371/journal.pone.0311484)
Supplement: S2 Table — AEs, adverse events; BCVA, best-corrected visual acuity; CST, central subfield thickness; DME, diabetic macular edema; ETDRS DRSS, Early Treatment Diabetic Retinopathy Study Diabetic Retinopathy Severity Scale; FA, fluorescein angiography; IRF, intraretinal fluid; NEI VFQ-25, The 25-item National Eye Institute Visual Function Questionnaire; OCT-A, optical coherence tomography angiography; Q24W, every 24 weeks; SD-OCT, spectral-domain optical coherence tomography; SRF, subretinal fluid; WPAI, Work Productivity and Activity Impairment Questionnaire. (PDF) [file pone.0311484.s002.pdf]

Supporting Information

Table S2. SWAN trial prespecified endpoints.

**Primary efficacy endpoint**
• Change in BCVA from baseline at 1 year (averaged over weeks 52, 56, and 60)
**Secondary efficacy endpoint**
• Change in BCVA from baseline at the visit proximate to the last dose among weeks 52, 56, and 60 (week 52 unless the dose is at week 52 or week 56 where it will then be week 56 or week 60, respectively)<br>• BCVA and BCVA change from baseline over time<br>• Proportions of patients with an ≥0.3 BCVA improvement from baseline over time<br>• Proportions of patients without an ≥0.3 BCVA worsening from baseline over time<br>• Proportions of patients who meet predefined thresholds for BCVA (i.e., decimal visual acuity ≥0.5, ≥0.7, ≥1.0, or ≤0.1) over time<br>• Proportion of patients over time with a BCVA better than before being diagnosed with DME<br>• Proportion of patients with a ≥2-step improvement in ETDRS-DRSS from baseline over time<br>• Proportion of patients with a ≥3-step improvement in ETDRS-DRSS from baseline over time<br>• Change in CST from baseline at 1 year (averaged over weeks 52, 56, and 60)<br>• CST and CST change from baseline over time

- Proportions of patients with absence of DME (Spectralis SD-OCT: CST <325 µm, or Cirrus SD-OCT or Topcon SD-OCT: CST <315 µm) over time
- Proportion of patients with IRF, and/or SRF over time
- Proportion of patients with an absence of IRF, and/or SRF over time
- Proportion of patients who develop new proliferative diabetic retinopathy over time
- Proportion of patients who develop new neovascular glaucoma over time
- Proportion of patients by faricimab treatment intervals
- Average number of faricimab doses administered
- NEI VFQ-25 composite score and change from baseline over time

Exploratory efficacy endpoints

- Proportion of patients achieving Q24W faricimab dosing
- Change from baseline in the ischemic nonperfusion area in the macular and the total retinal area (evaluated by FA and OCT-A) over time
- Change from baseline over time in vascular leakage area in the macular and the total retinal area (evaluated by FA)
- Proportion of patients without vascular leakage in the macula and the total retinal area (evaluated by FA) from baseline over time
- Change in the number of macular microaneurysms (evaluated by FA and OCT-A) from baseline over time
- Change in foveal avascular zone (evaluated by OCT-A) from baseline over time
- Change in vascular density in superficial capillary plexus and deep capillary plexus (evaluated by OCT-A) from baseline over time

- Disorganization of the retinal inner layers from baseline over time
- Hyperreflective foci from baseline over time
- Ellipsoid zone disruption from baseline over time
- Pathological changes of diabetic retinopathy from baseline over time
- Relationship between parameters at baseline and prognosis (BCVA and other endpoints such as dosing frequency of faricimab) from baseline over time
- Relationship between presence or absence of active DME (CST ≥325 μm on SD-OCT and clinically significant IRF/SRF, or CST ≥325 μm on SD-OCT and ≥315 μm on Cirrus or Topcon SD-OCT) at week 12 and prognosis (BCVA and other endpoints such as dosing frequency of faricimab)
- Relationship between anatomic parameters and BCVA
- Change from baseline in the Near Activities, Distance Activities, and Driving subscales of NEI VFQ-25 over time
- Proportion of patients with >4 points improvement in the NEI VFQ-25 composite score over time
- Relationship between BCVA and CST and quality of life (influence on work by test result or visit/dosing interval [WPAI])
- Imaging biomarkers using artificial intelligence
- Change in patient's subjective symptoms using the application for patients
- Usage situation and frequency of use of the application for patients, and frequency of appearance of words in texts entered

Safety endpoints (monitored between the first faricimab dose and the last trial visit [or withdrawal of consent or loss to follow-up])
• All AEs that occur between the first dose of faricimab and the last trial visit (or withdrawal of consent or loss to follow-up)<br>• Incidence and severity of ocular AEs<br>• Incidence and severity of nonocular AEs<br>• Blood pressure<br>• Heart rate

AEs, adverse events; BCVA, best-corrected visual acuity; CST, central subfield thickness; DME, diabetic macular edema; ETDRS DRSS, Early Treatment Diabetic Retinopathy Study Diabetic Retinopathy Severity Scale; FA, fluorescein angiography; IRF, intraretinal fluid; NEI VFQ-25, The 25-item National Eye Institute Visual Function Questionnaire; OCT-A, optical coherence tomography angiography; Q24W, every 24 weeks; SD-OCT, spectral-domain optical coherence tomography; SRF, subretinal fluid; WPAI, Work Productivity and Activity Impairment Questionnaire.
