# Supplementary material for: Optimization of individualized faricimab dosing for patients with diabetic macular edema: Protocol for the SWAN open-label, single-arm clinical trial
Source: PLoS One. 2024 Oct 10;19(10):e0311484. doi: 10.1371/journal.pone.0311484 (PMC11466402; doi:10.1371/journal.pone.0311484)
Supplement: S1 Fig — BCVA, best-corrected visual acuity; Q4W, every 4 weeks; Q8W, every 8 weeks; T&E, treat-and-extend. *, primary efficacy outcome: change in BCVA from baseline at 1 year, averaged over weeks 48, 52, and 56 (primary endpoint visits). (PDF) [file pone.0311484.s003.pdf]

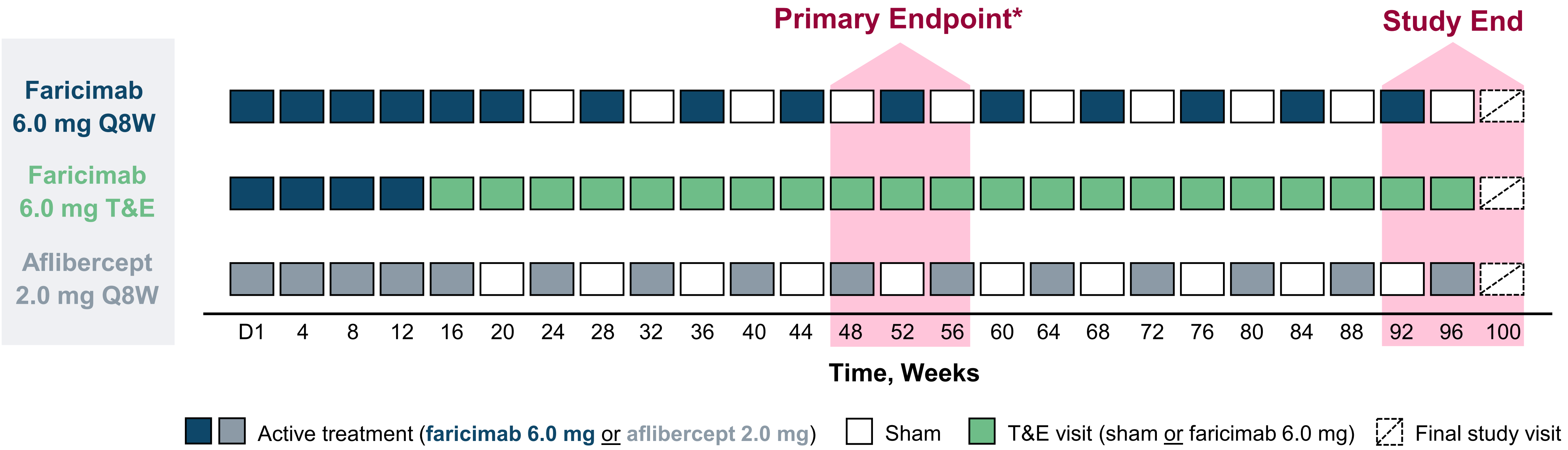
Primary Endpoint*
Study End
Faricimab
6.0 mg Q8W
Faricimab
6.0 mg T&E
Aflibercept
2.0 mg Q8W
D1
4
8
12
16
20
24
28
32
36
40
44
48
52
56
60
64
68
72
76
80
84
88
92
96
100
Time, Weeks
Active treatment (faricimab 6.0 mg or aflibercept 2.0 mg)
Sham
T&E visit (sham or faricimab 6.0 mg)
Final study visit
