# Supplementary material for: Optimization of individualized faricimab dosing for patients with diabetic macular edema: Protocol for the SWAN open-label, single-arm clinical trial
Source: PLoS One. 2024 Oct 10;19(10):e0311484. doi: 10.1371/journal.pone.0311484 (PMC11466402; doi:10.1371/journal.pone.0311484)
Supplement: S1 Protocol — (PDF) [file pone.0311484.s005.pdf]

# Clinical Trial Protocol

| | |
|---|---|
| Title: | **A MULTICENTER CLINICAL TRIAL TO INVESTIGATE OPTIMIZATION OF AN INDIVIDUALIZED DOSING REGIMEN OF FARICIMAB IN PATIENTS WITH DIABETIC MACULAR EDEMA IN THE CLINICAL SETTING** |
| Trial protocol Number: | CMA-0153 |
| Version: | Ver.2.0 |
| Investigational Medicinal Product: | Faricimab (genetical recombination) |
| Principal Investigator: | Department of Ophthalmology, Shinshu University School of Medicine<br>Professor, Toshinori Murata |
| Collaborative Institute: | Chugai Pharmaceutical Co., Ltd. |
| Research Secretariat: | Department of Ophthalmology, Shinshu University School of Medicine<br>Associate Professor, Takao Hirano |
| Supportive Secretariat: | IQVIA Services Japan G.K. |
| CRB Approval Date: | 2023/5/9 |

Table of Contents

Clinical Trial Protocol ... 1
Trial Protocol Synopsis ... 7
LIST OF ABBREVIATIONS AND DEFINITIONS OF TERMS ... 17
1. Background ... 19
1.1 Background on Diabetic Macular Edema ... 19
1.2 Background on Faricimab ... 19
1.3 Trial Rationale ... 19
2. Objectives and Endpoints ... 20
2.1 Objectives ... 20
2.2 Endpoints ... 20
2.2.1 Primary Endpoint ... 20
2.2.2 Secondary Endpoints ... 20
2.2.3 Exploratory Endpoints ... 21
2.2.4 Safety Endpoints ... 22
3. Trial Outline ... 23
3.1 Type of Trial ... 23
3.2 Trial Design ... 23
3.3 End of Trial and Length of Trial ... 24
3.4 Rationale for Trial Design ... 25
3.5 Rationale for Sample Size ... 25
3.6 Significance of Trial ... 26
3.7 Benefits and Risks Involved in Trial Participation ... 26
4. Subjects and Methods ... 28
4.1 Subjects ... 28
4.1.1 Inclusion Criteria ... 28
4.1.2 Exclusion Criteria ... 28
4.2 Enrollment ... 31
4.2.1 Enrollment Procedure ... 31
4.2.2 Issue and Notice of Enrollment Result ... 31
4.2.3 Enrollment in Multiple Trials ... 31
4.2.4 Precautions for Enrollment ... 31
4.2.5 Procedure for Enrollment Closes ... 32
4.3 Schedule of Activities ... 32
4.3.1 Informed Consent and Screening Log ... 32
4.3.2 Medical and Surgical History, and Background Information ... 32
4.3.3 Body Weight and Height ... 33

4.3.4 Vital Signs ..... 33
4.3.5 Ophthalmic Examination ..... 33
4.3.5.1 Finger-counting Test ..... 33
4.3.5.2 Intraocular Pressure ..... 33
4.3.5.3 Refraction Test ..... 33
4.3.5.4 Visual Acuity Test ..... 33
4.3.5.5 Slitlamp Microscopy ..... 34
4.3.5.6 Funduscopy ..... 34
4.3.5.7 Optical Coherence tomography (OCT) ..... 34
4.3.6 ETDRS Diabetic Retinopathy Severity Scale (ETDRS DRSS) ..... 35
4.3.7 Patient Reported Outcome ..... 35
4.3.7.1 NEI VFQ-25 ..... 35
4.3.7.2 WPAI ..... 36
4.3.7.3 Survey of patient's subjective symptoms ..... 36
4.3.7.4 Patient's problems in daily life, questions, etc. ..... 36
4.3.8 Clinical Laboratory Test ..... 36
4.3.9 Unscheduled Visit ..... 36
4.3.10 Patient Discontinuation from Trial ..... 37
4.3.11 Site Discontinuation ..... 37
4.3.12 Discontinuation of the Entire Research ..... 38
5. Treatment and Criteria for Treatment Change ..... 38
5.1 Investigational Medicinal Product (IMP) ..... 38
5.2 Treatment ..... 39
5.3 Criteria for Treatment Interruption/Discontinuation ..... 40
5.4 Concomitant and Supportive Therapy ..... 41
5.4.1 Prescribed Concomitant and Supportive Therapy ..... 41
5.4.2 Recommended/Unrecommended Concomitant and Supportive Therapy ..... 41
5.4.3 Permissible Concomitant and Supportive Therapy ..... 41
5.4.4 Prohibited Concomitant Drug and Therapy ..... 41
6. Safety Assessments ..... 42
6.1 Expected Adverse Events of Individual Drugs ..... 42
6.2 Definition of Adverse Event ..... 42
6.2.1 Definition of Adverse Event ..... 42
6.2.2 Definition of Adverse Reaction ..... 42
6.2.3 Other Adverse Events ..... 42
6.2.4 Seriousness of Adverse Event ..... 42
6.2.5 Severity of Adverse Event ..... 43
6.2.6 Criteria for Determination of Causal Relationship ..... 43

7. Adverse Event Reporting ... 44
7.1 Adverse Event Reporting Period ... 44
7.2 Actions When Any Adverse Event Occurs ... 44
7.3 Actions in Case of “Disease or Like” or Infection ... 44
7.4 Follow-up of Patients after Adverse Event ... 46
7.5 Pregnancy in Female Patients ... 46
7.6 Reporting of Overdose/Dosing Error ... 46
8. Statistical Considerations and Analysis Plan ... 46
8.1 Planned Number of Patients and Estimated Trial Period ... 46
8.2 Analysis Populations ... 47
8.3 Analysis of Primary Endpoint ... 47
8.4 Analysis of Secondary Endpoints ... 48
8.5 Analysis of Exploratory Endpoints ... 48
8.6 Interim Analysis ... 48
8.7 End of Trial ... 48
9. Collection and Management ... 48
9.1 Data Quality Assurance ... 48
9.2 Electronic Case Report Form (eCRF) ... 48
9.2.1 Input Items of eCRF ... 49
9.3 Identification of Source Data ... 49
9.3.1 Data Recorded Only in eCRF Only ... 49
9.3.2 Source Document ... 49
9.3.3 Use of Computerized Systems ... 49
9.4 Handling and Storage of Record, Information, and Sample ... 49
9.4.1 Trial site ... 49
9.4.2 Collaborative Institute ... 50
9.4.3 Supportive Secretariat ... 51
9.4.4 Imaging CRO ... 51
9.5 Record Retention and Destruction of Provision of Information ... 51
10. Ethical Considerations ... 52
10.1 Protection of Trial Participants ... 52
10.2 Informed Consent ... 52
10.2.1 Provision of Information and Consent ... 52
10.2.2 Withdrawal of Consent ... 53
10.3 Patient consultation ... 53
10.4 Protection of personal information and Identification of Patients ... 54
10.4.1 Purpose of Use of Personal Information, Information to be Used, and Usage .. 54
10.4.2 Secondary Use of Data ... 55

10.4.3 Response to a Request for Information Disclosure .......... 55
10.4.4 Information Management System .......... 55
10.5 Need and System for Genetic Counseling .......... 55
10.6 Compliance with Trial Protocol .......... 55
10.7 Approval by CRB and Notification to MHLW .......... 55
10.7.1 Procedure for New Application .......... 56
10.7.2 Procedure to be Performed by Investigators at Each Trial Site .......... 56
10.7.3 Approval of Trial Conduct at Each Trial Site .......... 56
10.8 Procedure for Application of Amendment .......... 57
10.8.1 Procedure to be Performed by Principal Investigator .......... 57
10.8.2 Procedure to be Performed by Investigators at Each Trial Site .......... 57
10.8.3 Review and Approval of Progress of Trial or Trial Continuation (Periodical Report) .......... 57
10.8.4 Reporting of Progress of Clinical Trial .......... 58
10.9 Conflict of Interest .......... 58
10.9.1 Managing Conflict of Interest Related to This Clinical Trial .......... 58
10.9.2 Funding Source of Clinical Trial/Funding, and Financial Relationships .......... 59
10.9.3 Information Disclosure .......... 59
10.10 Trial Expense .......... 59
10.10.1 Expense for Treatment .......... 59
10.10.2 Compensation for Health Damage .......... 60
11. Record、Monitoring, and Audit of Trial .......... 60
11.1 Record of Trial .......... 60
11.2 Monitoring .......... 60
11.2.1 Central Monitoring .......... 60
11.2.2 On-site Monitoring .......... 61
11.2.3 Audit .......... 61
11.3 Violation and Deviation from Trial Protocol .......... 61
12. Publication of Data .......... 61
12.1 Record to the Public Database .......... 62
12.2 Publication of Data .......... 62
12.3 Layperson Summary (LPS) .......... 63
13. Discontinuation, Interruption, or Termination of Entire Trial .......... 63
13.1 End of Entire Trial .......... 63
13.2 Discontinuation or Interruption of Entire Trial .......... 63
13.3 Clinical Trial Report .......... 63
13.4 Procedure at the End of Trial .......... 63
14. Attribution of Trial Results .......... 64

15. Trial Structure ..... 64
15.1 Principal Investigator ..... 64
15.2 Responsible Institute/Collaborative Institute ..... 64
15.2.1 Representative Director of Responsible Institute/Collaborative Institute ..... 64
15.2.2 Division Director of Responsible Institute/Collaborative Institute ..... 64
15.2.3 Medical Expert and Responsible Person in Responsible Institute/Collaborative Institute ..... 65
15.3 Sponsor ..... 66
15.4 Research Executive Committee (order of the Japanese syllabary) ..... 66
15.5 Research Secretariat ..... 66
15.6 Trial sites ..... 67
15.7 Responsible Statistician ..... 67
15.8 Supportive Secretariat ..... 67
15.9 Organization Responsible for Monitoring ..... 67
15.10 Organization Responsible for Data Management ..... 67
15.11 Organization Responsible for Audit ..... 68
15.12 Person Supporting Research & Development Plan ..... 68
15.13 Person in Charge of Coordination and Management ..... 68
15.14 Imaging CRO ..... 68
15.15 ePRO Vendor ..... 68
16. Reference ..... 69

List of Appendices

Appendix 1 Schedule of Activities ..... 71
Appendix 2 Entry Items in eCRF ..... 74

Annex 1 List of Trial Sites and Investigators
Annex 2 Investigation report form on overdose/medication error
Annex 3 Investigation report form on pregnancy case
Annex 4 Investigation report form on a newborn child

# Trial Protocol Synopsis

| | |
|---|---|
| Title: | A Multicenter Clinical Trial to Investigate Optimization of an Individualized Dosing Regimen of Faricimab in Patients with Diabetic Macular Edema in the Clinical Setting |
| Trial protocol Number: | CMA-0153 |
| Version: | Ver. 2.0 |
| Investigational Medicinal Product: | Faricimab (genetical recombination) |
| Condition: | Diabetic Macular Edema |
| Responsible Research Institute: | Chugai Pharmaceutical Co., Ltd. |

I Objectives and Endpoints

(1) Objectives

The objective of this trial is to assess the efficacy and durability of the efficacy of faricimab in the maintenance phase when administered to patients with diabetic macular edema (DME) using a dosing regimen adapted to clinical practice, and to explore the characteristics of the patient population for whom the dosing interval can be extended.

(2) Endpoints

① Primary Endpoint

Change from baseline in best-corrected visual acuity (BCVA) at 1 year*. The BCVA is measured as decimal visual acuity and converted to logarithm of the minimum angle of resolution (logMAR) values to calculate the changes in BCVA.

* “1 year” means the average of Weeks 52, 56, and 60.

② Secondary Endpoints

- Change from baseline in BCVA at a visit proximate to the last dose among W52, W56, and W60** (logMAR).
  ** i.e., W56 and W60, respectively, if the last dose occurs at W52 or W56. Otherwise, W52.
- Change from baseline in central subfield thickness (CST) at 1 year.

The following items at each time points

- BCVA and change from baseline in BCVA (logMAR)
- Proportion of patients with a ≥ logMAR 0.3 improvement from baseline in BCVA
- Proportion of patients without a ≥ logMAR 0.3 worsening from baseline in BCVA
- Proportion of patients with BCVA (decimal visual acuity) ≥ 0.5
- Proportion of patients with BCVA (decimal visual acuity) ≥ 0.7
- Proportion of patients with BCVA (decimal visual acuity) ≥ 1.0
- Proportion of patients with BCVA (decimal visual acuity) ≤ 0.1
- Proportion of patients with visual acuity better than that before getting DME***

*** If visual acuity before getting DME is not recorded in the medical record, patient will be inquired for it.

- CST and change from baseline in CST
- Proportion of patients with absence of DME (CST < 325 μm [< 315 μm depending on the machine]) DME
- Proportion of patients with absence of intraretinal fluid (IRF), proportion of patients with absence of subretinal fluid (SRF), and proportion of patients with absence of both IRF and SRF.
- Proportion of patients with presence of IRF, proportion of patients with presence of SRF, and proportion of patients with presence of both IRF and SRF.
- Proportion of patients with a ≥ 2-step diabetic retinopathy severity (DRS) improvement from baseline on the Early Treatment Diabetic Retinopathy Study (ETDRS) Diabetic Retinopathy Severity Scale (DRSS)
- Proportion of patients with a ≥ 3-step DRS improvement from baseline on the ETDRS DRSS
- Proportion of patients who develop new PDR
- Proportion of patients who develop new neovascular glaucoma
- Proportion of patients by treatment intervals
- Average number of dosing of faricimab
- The 25-item National Eye Institute Visual Function Questionnaire (NEI VFQ-25) composite score and its change from baseline

③ Exploratory Endpoints

- Change from baseline in the chemic non-perfusion area in the macular and the total retinal area over time (evaluated by fluorescein angiography [FA] and optical coherence tomography-angiography [OCT-A] )
- Change from baseline in vascular leakage area in the macula and the total retinal area over time (evaluated by FA)
- Proportion of patients without vascular leakage in the macula and the total retinal area over time (evaluated by FA)
- Change from baseline in numbers of microaneurysm in the macula (evaluated by FA and OCT-A)
- Change from baseline in foveal avascular zone (FAZ) over time (evaluated by OCT-A)
- Change from baseline in vascular density in superficial capillary plexus (SCP) and deep capillary plexus (DCP) over time (evaluated by OCT-A)
- Evaluation of the following items over time
  - ➢ Disorganization of the retinal inner layers (DRIL)
  - ➢ Hyperreflective foci
  - ➢ Ellipsoid Zone (EZ) Disruption
- Pathological changes of DME
- Relationship between parameters at baseline and the prognosis (BCVA and other endpoints such as dosing frequency of IMP)
- Relationship between presence or absence of active DME**** at W12 and the prognosis (BCVA and the other endpoints such as dosing interval of the IMP)

**** DME is regarded as active if CST is ≥ 325 μm***** and clinically significant****** IRF or SRF is observed.

***** ≥ 325 μm with Spectralis SD-OCT and ≥ 315 μm with Cirrus or Topcon SD-OCT (or other equivalent OCT)

****** The condition is considered clinically significant if it is deemed to be a cause of vision loss or other aggravation of the disease.

- Relationship between anatomical parameters and visual acuity
- Change from baseline in Near Activities, Distance Activities, and Driving subscales of NEI VFQ-25 at each time points
- Proportion of patients with ≥ 4 points improvement in NEI VFQ-25 composite score at each time points
- Relationship between the numerical values (e.g., BCVA and CST) and the quality of life (QOL) data: influence on work by test result or visit/dosing interval (Work Productivity and Activity Impairment Questionnaire [WPAI])
- Exploratory search for imaging biomarkers using artificial intelligence (AI)
- Change in patient's subjective symptoms assessed by the application for patients
- Usage status and input frequency of the application for patients, and frequency of appearance of words in entered texts
- Proportion of patients who achieve Q24W during the research period

④ Safety Endpoints

- Incidence and severity of ocular adverse events
- Incidence and severity of non-ocular adverse events
- Vital signs (blood pressure and pulse rate)

II Trial Design

This is an open-label, single-arm, interventional, multicenter trial to evaluate the efficacy and safety of a new dosing regimen of faricimab adapted to clinical practice in patients with DME. This trial is a specified clinical trial subject to the Clinical Trials Act.

Patients participating in the trial will receive intravitreal administration of faricimab 6.0 mg for 2 years. In the initial phase, all patients will receive faricimab once every 4 weeks (Q4W) for 4 consecutive doses. However, the $4^{th}$ dosing may be skipped depending on the disease activity*. The dosing schedule from W12 onwards will be determined based on the DME activity* after W12.

Patients without active DME* at W12 and thereafter will be treated with the following regimen (Figure 1.1-1, Extension-by-8W Regimen).

A) Injection Interval

Dosing intervals will be extended in an 8-week increments up to a maximum of once every 24 weeks (Q24W).

B) Visit Interval

Patients will visit trial sites every 4 weeks up to W24, and then every 8 weeks depending on a dosing interval. In addition, patients will visit trial sites also at W52 and W60 for the trial assessments.

If active DME is observed at W12 or subsequent visits, the patient will be transferred to the following regimen thereafter. (Figure 1.1-1, Extension-by-4W Regimen).

A) Injection Interval
At a visit when active DME is observed, the patient will receive faricimab within the day. Then patients will receive faricimab at an interval 4 weeks less than the time between the last dose and the visit date when active DME is observed. However, the minimum dosing interval should be once every 8 weeks (Q8W). Thereafter, unless active DME is observed, the next and subsequent dosing intervals should be extended in a 4-week increments. The maximum dosing interval should be Q24W.

B) Visit Interval
Patients will visit trial sites every 4 weeks up to W24, and then every 8 weeks. In addition, patients will visit trial sites also at W52 and W60 for the trial assessments. In addition, patients will visit trial sites also at W52, W56, W60, and W112 for the trial assessments.

* DME is regarded as active if CST is ≥ 325 μm** and clinically significant*** IRF or SRF is observed. Determination of disease activity will be performed for the study eye only.
** ≥ 325 μm with Spectralis SD-OCT and ≥ 315 μm with Cirrus or Topcon SD-OCT (or other equivalent OCT)
*** The condition is considered clinically significant if it is deemed to be a cause of vision loss or other aggravation of the disease.

This trial will be conducted in compliance with the “Declaration of Helsinki” (translated by the Japan Medical Association), “Clinical Trials Act” (Act No. 16, 2017), “Ordinance for Enforcement of the Clinical Trials Act” (Ministerial Ordinance No.17, Ministry of Health, Labour and Welfare, 2018), “Ethical Guidelines for Medical and Health Research Involving Human Subjects” (Notification No. 1, Ministry of Education, Culture, Sports, Science and Technology, Ministry of Health, Labour and Welfare, and Ministry of Economy, Trade and Industry, March 23, 2021, partial revision on March 10, 2022).

Figure 1.1-1 Dosing Schedule

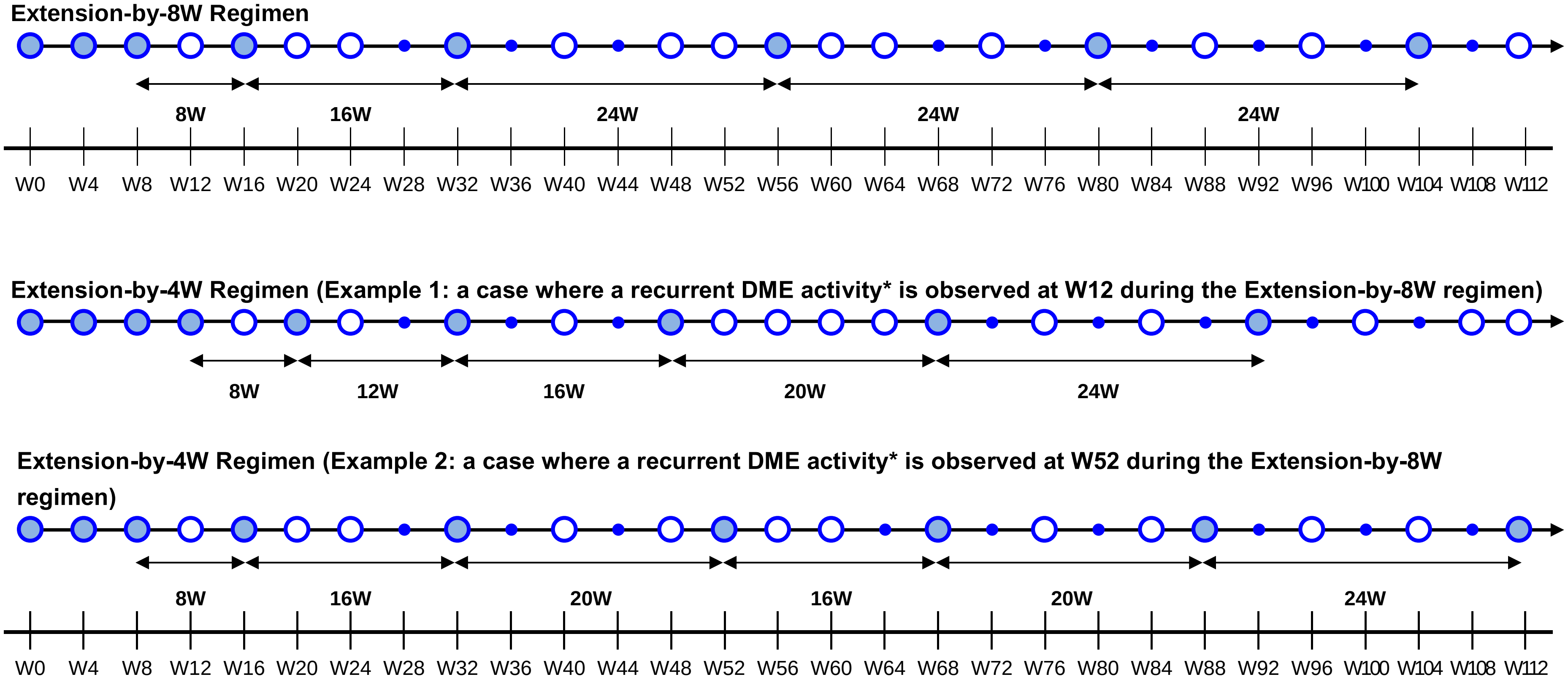


●: Visit (with dosing), ○: Visit (without dosing), •: No visit

* DME is regarded as active if CST is ≥ 325 µm** and clinically significant*** IRF or SRF is observed. Determination of disease activity will be performed for the study eye only.

** ≥ 325 µm with Spectralis SD-OCT and ≥ 315 µm with Cirrus or Topcon SD-OCT (or other equivalent OCT)

*** The condition is considered clinically significant if it is deemed to be a cause of vision loss or other aggravation of the disease.

III Planned Number of Patients

70 patients

IV Target Population

Subjects who meet all of the following inclusion criteria and do not meet all of the following exclusion criteria will be included in this trial.

(1) Inclusion Criteria

General Inclusion Criteria

① Patients who provide written informed consent

② Age ≥ 18 at the time of consent

③ Confirmed diagnosis of diabetes mellitus (Type 1 or Type 2)

④ Ability and willingness to undertake all scheduled visits and assessments

Inclusion Criteria for Study Eye

One eye will be designated as a study eye. If both eyes are determined to be eligible, the eye with the worse BCVA at screening will be selected as a study eye, unless the investigator or co-investigator determines that the other eye is more appropriate for research treatment.

① Macular thickening secondary to DME involving the center of the fovea with CST ≥ 325 μm, as measured on Spectralis SD-OCT, or ≥ 315 μm, as measured on Cirrus SD-OCT or Topcon SD-OCT (or other equivalent OCTs) at screening

② BCVA of 0.0625 ~ 0.7 (decimal visual acuity) on visual acuity test conducted at screening

③ Sufficiently clear optic media and adequate pupillary dilatation to allow acquisition of good quality color fundus photography (CFP) and other imaging modalities

(2) Exclusion Criteria

General Exclusion Criteria

① History of severe allergic reaction or anaphylactic reaction to a biologic agent or known hypersensitivity to faricimab and any of its excipients, mydriatic eye drops, anesthetics or antimicrobials

② History of other disease, other non-diabetic metabolic dysfunction, physical examination finding, historical or current clinical laboratory finding giving reasonable suspicion of a condition that contraindicates the use of the faricimab or that might affect interpretation of the results of the trial or renders the patient at high risk for treatment complications in the opinion of the investigator or co-investigator

③ Active cancer within the past 12 months except for appropriately treated carcinoma in situ of the cervix, non-melanoma skin carcinoma, and prostate cancer with a Gleason score of ≤ 6 and a stable prostate-specific antigen for > 12 months

④ Systemic treatment for suspected or active systemic infection

⑤ Participation in an investigational study that involves treatment with any drug or device (except vitamins and minerals) within 3 months prior to Day 1

⑥ Administration of systemic pro-angiogenic treatments for the peripheral or coronary ischemia (e.g., limb ischemia or myocardial infarction) within 3 months prior to Day 1

⑦ Not willing to comply with the trial or follow-up procedures

⑧ Renal failure requiring renal transplant, hemodialysis, or peritoneal dialysis or anticipated to require hemodialysis or peritoneal dialysis at any time during the trial

⑨ Uncontrolled blood pressure (defined as systolic >180 mmHg and/or diastolic >100 mmHg while a patient is at rest). If a patient's initial reading exceeds these values, a second reading may be obtained later the same day or on another day during the screening period.

⑩ Stroke (cerebral vascular accident) or myocardial infarction within 6 months prior to Day 1

⑪ Pregnancy or breastfeeding, or intention to become pregnant during the trial

⑫ Women of childbearing potential* who does not agree to remain abstinent (refrain from heterosexual intercourse) or use acceptable contraceptive methods that result in a failure rate of < 1% per year** during the treatment period and for at least 38 days after the final dose of the IMP.

* Postmenarchall women who have not reached postmenopausal status (amenorrhea for at least 12 consecutive months with no cause other than menopause), and are not permanently infertile by surgery (removal of ovaries, fallopian tubes and/or uterus) or other causes as determined by the investigator or co-investigator (e.g., Müllerian duct dysplasia) considered women of child-bearing potential. According to this provision, women with unilateral tubal ligation are considered women of child-bearing potential.

** Examples of contraceptive methods with annual failure rates of less than 1% include bilateral tubal ligation, male sterilization, hormonal contraceptives that inhibit ovulation, hormone-releasing intrauterine devices, and copper-added intrauterine devices. The reliability of sexual abstinence should be evaluated with respect to the duration of the clinical research and each patient's preferences and normal lifestyle. Cyclic abstinence (calendar, ovulation day, symptomatic temperature, post-ovulation, etc.) and external ejaculation are not adequate contraceptive methods.

⑬ Requirement for continuous use of any prohibited concomitant medications or therapies listed in Section 5.4.4.

⑭ Patients who are not receiving treatment for diabetes.

Exclusion Criteria for Study Eye

① High-risk PDR in the study eye (using any of the following established criteria for high-risk PDR)

1) Any vitreous or pre-retinal hemorrhage
2) Neovascularization elsewhere ≥1/2 disc area within an area equivalent to the mydriatic ETDRS 7 fields on clinical examination or CFPs
3) Neovascularization at disc ≥1/3 disc area on clinical examination

② Tractional retinal detachment, pre-retinal fibrosis, vitreomacular traction syndrome, or epiretinal membrane involving the fovea or disrupting the macular architecture in the study eye

③ Active rubeosis

④ Uncontrolled glaucoma

⑤ History of retinal detachment or macular hole (Stage 3 or 4)

⑥ Aphakia or implantation of anterior chamber intraocular lens

⑦ Intravitreal administration of anti-VEGF agents within 3 months prior to Day 1 (applicable to patients whose study eyes were previously treated with intravitreal anti-VEGF agents), or any intravitreal administration of anti-VEGF agents to study eye prior to Day 1

(applicable for treatment-naive patients). Enrollment of patients who have a medication history of intravitreal administration of anti-VEGF agents should be no more than 25% of the total.

⑧ History of panretinal photocoagulation (PRP), macular laser (focal, grid, or micropulse), any cataract surgery or treatment for complications of cataract surgery with steroids or yttrium-aluminum-garnet (YAG) laser capsulotomy within 3 months prior to Day 1

⑨ Any other intraocular surgery (e.g., corneal transplantation, glaucoma filtration, pars plana vitrectomy, corneal transplant, or radiotherapy)

⑩ Any intravitreal or periocular (subtenon) corticosteroid treatment within 6 months prior to Day 1

⑪ Treatment for other retinal diseases that can lead to macular edema

⑫ Prior intravitreal administration of faricimab in either eye

Exclusion Criteria for Fellow (non-study) Eye

① Non-functioning non-study eye, defined as either:
  1) BCVA of hand motion or worse
  2) No physical presence of non-study eye (i.e., monocular)

Exclusion Criteria for Both Eyes

① Any history of idiopathic or immune-mediated uveitis in either eye

② Active ocular inflammation or suspected or active ocular or periocular infection in either eye on Day 1

Exclusion Criteria Related to Concurrent Ocular Conditions

① Any current or history of ocular disease other than DME that may confound the assessment of the macula or affect central vision in the study eye (choroidal neovascularization, age-related macular degeneration, retinal vein occlusion, uveitis, angioid streaks, histoplasmosis, active or inactive cytomegalovirus, pathological myopia, retinal detachment, retinal embolus, macular traction, macular hole, and other)

② Any current ocular condition that, in the opinion of the investigator or co-investigator, is currently causing or could be expected to contribute to irreversible vision loss due to a cause other than DME in the study eye (e.g., foveal atrophy, foveal fibrosis, pigment abnormalities, dense subfoveal hard exudates, or other non-retinal conditions)

IV End of Trial and Trial Period

The completion date of this clinical trial shall be the date when a summary of the clinical trial report is registered and published in the Japan Registry for Clinical Trials (jRCT).

VI Investigational Medicinal Product

Generic Name: faricimab (genetical recombination)
Brand Name: VABYSMO® solution for intravitreal injection

VII Schedule of Assessments

Refer to Appendix 1.

## VIII Statistical Methods

### (1) Primary Analysis

① Determination of Sample Size

For the primary endpoint of change from baseline in BCVA at 1 year after treatment initiation, assuming a true value of 0.22 [logMAR] and a standard deviation of 0.22 [logMAR] based on the phase 3 clinical study results of faricimab (YOSEMITE and RHINE studies), 45 cases will provide ≥ 80% probability that the point estimate of the primary endpoint is not more than 0.04 [logMAR] lower than the true value. In addition, assuming that the proportion of patients with a dosing interval of once every 16 weeks (Q16W) or more is 50% based on the clinical study results, the number of patients (23 cases) will provide ≥ 80% probability that the point estimate is not more than 0.04 [logMAR] lower the true value in that population, and the 95% confidence interval is 4.48 letters. This is less than 0.09 [logMAR] (one ETDRS line), which is considered clinically significant. Dropout rates at 1 year in YOSEMITE and RHINE studies were approximately 10%. However, the sample size will be 70 assuming a 35% dropout rate in the first year since patients bear the drug cost and patient's burden of visit is heavy in this research.

② Analysis of Primary Endpoint

The primary efficacy endpoint in this trial is the change from baseline in BCVA at 1 year after treatment initiation. The BCVA is expressed in logMAR value converted from decimal visual acuity and is analyzed using a mixed effect model for repeated measure (MMRM) model. The model includes visits (categorical variable) and baseline BCVA (continuous variable) as fixed effects and assumes an unstructured covariance structure for modeling of within-patient errors. For the mechanism of missing data, missing at random (MAR) will be assumed. The average of BCVA values at W52, W56, and W60 will be calculated.

### (2) Interim Analysis

Some analyses including the primary analysis will be performed when BCVA data at 1 year after treatment initiation are available for all patients.

## IX Contact Information

Principal Investigator: Shinshu University Hospital, Ophthalmology
Professor, Toshinori Murata

Research Secretariat: Shinshu University Hospital, Ophthalmology
Associate Professor, Takao Hirano
3-1-1 Asahi, Matsumoto Nagano 390-8621
Tel. 0263-37-2789

Collaborative Institute: Chugai Pharmaceutical Co., Ltd.
2-1-1 Nihonbashi Muromachi, Chuo-ku, Tokyo 103-8324
Tel. 03-3273-0866

Supportive Secretariat: IQVIA Services Japan G.K.
Keikyu Dai-1 Building 4-10-18 Takanawa, Minato-ku, Tokyo 108-0074

Tel. 03-6859-9500

LIST OF ABBREVIATIONS AND DEFINITIONS OF TERMS

| Abbreviation | Definition (English) | Definition (Japanese) |
|---|---|---|
| AI | artificial intelligence | 人工知能 |
| Ang-2 | angiopoietin-2 | アンジオポエチン-2 |
| BCVA | best corrected visual acuity | 最高矯正視力 |
| CFP | color fundus photograph | カラー眼底写真 |
| CRO | contract research organization | 開発業務受託機関 |
| CRB | certified review board | 認定臨床研究審査委員会 |
| CST | central subfield thickness | 中心領域網膜厚 |
| DME | diabetic macular edema | 糖尿病黄斑浮腫 |
| DR | diabetic retinopathy | 糖尿病網膜症 |
| DRIL | disorganization of the retinal inner layers | |
| DRS | diabetic retinopathy severity | 糖尿病網膜症の重症度 |
| eCRF | electronic case report form | 電子症例報告書 |
| EDC | electronic data capture | 電子データ収集 |
| ePRO | electronic patient-reported outcome | 電子的患者報告アウトカム |
| ETDRS | Early Treatment Diabetic Retinopathy Study | — |
| ETDRS DRSS | Early Treatment Diabetic Retinopathy Study Diabetic Retinopathy Severity Scale | — |
| EZ | ellipsoid zone | |
| FA | fluorescein angiography | フルオロセイン蛍光眼底造影 |
| Fab | fragment antigen-binding | — |
| FcRn | neonatal Fc receptor | 胎児性 Fc 受容体 |
| FAS | full analysis set | 最大の解析対象集団 |
| HbA1c | hemoglobin A1c | ヘモグロビン A1c |
| ICGA | Indocyanine green angiography | インドシアニングリーン蛍光眼底造影 |
| IgG | immunoglobulin G | 免疫グロブリン G |
| IRF | intraretinal fluid | 網膜内滲出液 |
| jRCT | Japan Registry of Clinical Trials | 認定臨床研究審査委員会申請・情報公開システム |
| logMAR | logarithm of the minimum angle of resolution | — |
| MAR | missing at random | ランダムな欠測 |
| MMRM | mixed effect model for repeated measure | 経時測定データに対する混合効果モデル |
| NEI VFQ-25 | The 25-item National Eye Institute Visual Function Questionnaire | — |
| NPDR | non-proliferative diabetic retinopathy | 非増殖糖尿病網膜症 |
| OCT | optical coherence tomography | 光干渉断層撮影 |

| | | |
|---|---|---|
| OCT-A | optical coherence tomography-angiography | OCT アンギオグラフィー |
| PDR | proliferative diabetic retinopathy | 増殖糖尿病網膜症 |
| PPS | Per-protocol set | Per-protocol 集団 |
| PRN | pro re nata | — |
| PRP | pan-retinal photocoagulation | 汎網膜光凝固術 |
| PTI | personalized treatment interval | — |
| Q4W | once every 4 weeks | 4週ごとに1回 |
| Q8W | once every 8 weeks | 8週ごとに1回 |
| Q16W | once every 16 weeks | 16週ごとに1回 |
| Q24W | once every 24 weeks | 25週ごとに1回 |
| QOL | quality of life | 生活の質 |
| SD | standard deviation | 標準偏差 |
| SD-OCT | spectral-domain optical coherence tomography | スペクトラルドメイン光干渉断層撮影 |
| SRF | subretinal fluid | 網膜下液 |
| TAE | treat-and-extend | — |
| VEGF | vascular endothelial growth factor | 血管内皮増殖因子 |
| VEGF-A | vascular endothelial growth factor A | |
| WPAI | Work Productivity and Activity Impairment Questionnaire | — |
| YAG | Yttrium Aluminum Garnet | イットリウムーアルミニウムーガーネット |

# 1. Background

## 1.1 Background on Diabetic Macular Edema

DME occurs in the background of diabetic retinopathy (DR), and involves edema caused by leakage of plasma into the macula[1]. DME is the leading cause of blindness in working-age populations in many industrialized countries and the most common vision-threatening complication of DR[2]. In the early stages of the disease, patients may have only metamorphopsia without loss of vision, but long-term morbidity causes prolonged macular edema, and complications of macular ischemia, atrophy of retinal pigment epithelial cells in the macula, and tractional retinal detachment to the macula due to proliferative changes, leading to severe vision loss[3]. The prevalence of DR among patients with diabetes in Japan is estimated to be 15% to 23% based on the Hisayama Study reported in 2004[4] and the Funagata Study reported in 2008[5]. Based on the results of the Hisayama Study and the Funagata Study, as well as data from the Ministry of Health, Labour and Welfare, it is estimated that there are approximately 5 million patients with DR, and approximately 1.1 million patients with PDR or DME in Japan[6].

The purpose of treatment of DME is to maintain or improve vision. For systemic management of DME, control of blood glucose, blood pressure, blood lipids, and renal function in line with the goals of diabetes treatment is important at first. The first-line treatment in the treatments of DME involving the central fovea is focal laser photocoagulation for focal macular edema with obvious leakage points, intravitreal anti-VEGF agents for diffuse macular edema, and vitrectomy for macular edema with obvious vitreoretinal traction[7].

## 1.2 Background on Faricimab

Faricimab (genetical recombinant) (hereinafter referred to as “faricimab”) is a novel humanized bispecific immunoglobulin G1 antibody developed by F. Hoffmann-La Roche, Ltd (hereinafter referred to as Roche ). Faricimab selectively binds to vascular endothelial growth factor A (VEGF-A) and angiopoietin-2 (Ang-2). One Fab domain binding to VEGF-A binds to VEGF-A with high affinity, and another Fab domain binding to Ang-2 binds to Ang-2 with high affinity and high selectivity. The Fc domain of faricimab was modified to prevent binding to the Fcγ receptor and embryonic Fc receptor (FcRn) on effector cells. The efficacy and safety of faricimab were confirmed in clinical studies in patients with neovascular age-related macular degeneration and DME. Based on these clinical study results, faricimab has been approved in Japan, the United States, Canada, EU, United Kingdom, Switzerland, Australia, Thailand and Singapore[8].

## 1.3 Trial Rationale

For the treatment of DME involving the central fovea, intravitreal injection of anti-VEGF agents has been the first choice of pharmacotherapy[7]. Anti-VEGF agents usually require long-term treatment, and various dosing regimens including fixed-dose, *pro re nata* (PRN) regimen, and treat-and-extend (TAE) regimen have been investigated to determine the optimal treatment interval[9][10]. However, in real-world clinical practice, a high percentage of patients drop out of treatment during the long-term treatment period, and it has become clear that the actual treatment outcomes are poorer than those observed in clinical studies[11][12]. One factor contributing to poor adherence to treatment with anti-VEGF agents is the high financial and psychological burden on patients due to the frequent dose[13][14]. Therefore, there is a need to

investigate new dosing regimens with less patient burden and factors related to dosing regimens and their efficacy[15]. This trial will investigate a new TAE dosing regimen of faricimab that more closely resembles the real-world dosing regimen for anti-VEGF agents. If the efficacy and safety of this dosing regimen are confirmed, it is expected to enable anti-VEGF therapy with less patient burden and fill the current clinical gap.

# 2. Objectives and Endpoints

## 2.1 Objectives

The objective of this trial is to assess the efficacy and durability of the efficacy of faricimab in the maintenance phase when administered to patients with DME using a dosing regimen adapted to clinical practice, and to explore the characteristics of the patient population for whom the dosing interval can be extended.

## 2.2 Endpoints

### 2.2.1 Primary Endpoint

Change from baseline in BCVA at 1 year*. The BCVA is measured as decimal visual acuity and converted to logMAR to calculate the changes in BCVA.
“1 year” means the average of Weeks 52, 56, and 60.

### 2.2.2 Secondary Endpoints

- Change from baseline in BCVA at a visit proximate to the last dose** among W52, W56, and W60 (logMAR)
  **i.e., W56 and W60, respectively, if the last dose occurs at W52 or W56. Otherwise, W52.
- Change from baseline in CST at 1 year

The following items at each time points

- BCVA and change from baseline in BCVA (logMAR)
- Proportion of patients with a ≥ logMAR 0.3 improvement from baseline in BCVA
- Proportion of patients without a ≥ logMAR 0.3 worsening from baseline in BCVA
- Proportion of patients with BCVA (decimal visual acuity) ≥ 0.5
- Proportion of patients with BCVA (decimal visual acuity) ≥ 0.7
- Proportion of patients with BCVA (decimal visual acuity) ≥ 1.0
- Proportion of patients with BCVA (decimal visual acuity) ≤ 0.1
- Proportion of patients with visual acuity better than that before getting DME***
  *** If visual acuity before getting DME is not recorded in the medical record, patient will be inquired for it.
- CST and change from baseline in CST
- Proportion of patients with absence of DME (CST < 325 μm [< 315 μm depending on the machine]) DME
- Proportion of patients with absence of IRF, patients with absence of SRF, and patients with absence of both IRF and SRF.
- Proportion of patients with presence of IRF, patients with presence of SRF, and patients with presence of both IRF and SRF.
- Proportion of patients with a ≥ 2-step DRS improvement from baseline on the ETDRS-DRSS
- Proportion of patients with a ≥ 3-step DRS improvement from baseline on the ETDRS DRSS
- Proportion of patients who develop new PDR

- Proportion of patients who develop new neovascular glaucoma
- Proportion of patients by treatment intervals
- Average number of dosing of faricimab
- The NEI VFQ-25 composite score and its change from baseline

2.2.3 Exploratory Endpoints

- Change from baseline in the ischemic non-perfusion area in the macular and the total retinal area over time (evaluated by FA and OCT-A*)
- Change from baseline in vascular leakage area in the macula and the total retinal area over time (evaluated by FA)
- Proportion of patients without vascular leakage in the macula and the total retinal area over time (evaluated by FA)
- Change from baseline in numbers of microaneurysm in the macula (evaluated by FA and OCT-A)
- Change from baseline in FAZ over time (evaluated by OCT-A)
- Change from baseline in vascular density in SCP and DCP over time (evaluated by OCT-A)
- Evaluation of the following items over time
    - DRIL
    - Hyperreflective foci
    - EZ disruption
- Pathological changes of DR
- Relationship between parameters at baseline and the prognosis (BCVA and other endpoints such as dosing frequency of IMP)
- Relationship between presence or absence of active DME* at W12 and the prognosis (BCVA and other endpoints such as dosing interval of the IMP)

  * DME is regarded as active if CST is ≥ 325 μm** and clinically significant*** IRF or SRF is observed. Determination of disease activity will be performed for the study eye only.

  ** ≥ 325 μm with Spectralis SD-OCT and ≥ 315 μm with Cirrus or Topcon SD-OCT (or other equivalent OCT)

  *** The condition is considered clinically significant if it is deemed to be a cause of vision loss or other aggravation of the disease.
- Relationship between anatomical parameters and visual acuity
- Change from baseline in Near Activities, Distance Activities, and Driving subscales of NEI VFQ-25 at each time points
- Proportion of patients with ≥ 4 points improvement in NEI VFQ-25 composite score at each time points
- Relationship between the numerical values (e.g., BCVA and CST) and the QOL data: influence on work by test result or visit/dosing interval (WPAI)
- Exploratory search for imaging biomarkers using AI
- Change in patient's subjective symptoms assessed by the application for patient
- Usage status and input frequency of the application for patients, and frequency of appearance of words in entered texts
- Proportion of patients who achieve Q24W during the research period

### 2.2.4 Safety Endpoints

- Incidence and severity of ocular adverse events
- Incidence and severity of non-ocular adverse events
- Vital signs (blood pressure and pulse rate)

# 3. Trial Outline

## 3.1 Type of Trial

This is an open-label, single-arm, multicenter clinical trial with interventions, in which the presence or absence and extent of testing, medication, and medical practice for diagnostic or therapeutic purpose are controlled for research purpose. This trial is a specified clinical trial subject to the Clinical Trials Act.

This trial will be conducted in compliance with the "Declaration of Helsinki" (translated by the Japan Medical Association)*, the "Clinical Trials Act" (Act No. 16, 2017)**, "Ordinance for Enforcement of Clinical Trials Act" (Ministerial Ordinance No. 17, Ministry of Health, Labor and Welfare of 2008)**, "Ethical Guidelines for Medical and Health Research Involving Human Subjects" (Notification No. 1, Ministry of Education, Culture, Sports, Science and Technology, Ministry of Health, Labor and Welfare, Ministry of Economy, Trade and Industry, March 23, 2021, partial revision on March 10, 2022)***, related notices, and this trial protocol.

* http://dl.med.or.jp/dl-med/wma/helsinki2013j.pdf
** http://www.mhlw.go.jp/stf/seisakunitsuite/bunya/0000163417.html
*** https://www. mhlw.go.jp/content/000909926.pdf

## 3.2 Trial Design

This is an open-label, single-arm, interventional, multicenter trial to evaluate the efficacy and safety of a new dosing regimen of faricimab adapted to clinical practice in patients with DME. This trial is a specified clinical trial subject to the Clinical Trials Act.

Patients participating in the trial will receive intravitreal administration of faricimab 6.0 mg for 2 years. In the initial phase, all patients will receive faricimab Q4W for 4 consecutive doses. However, the $4^{th}$ dosing may be skipped depending on the disease activity*. The dosing schedule from W12 onwards will be determined based on the DME activity* after W12.

Patients without active DME* at W12 and thereafter will be treated with the following regimen (Figure 1.1-1, Extension-by-8W Regimen).

A) Injection Interval

Dosing intervals will be sequentially extended in an 8-week increments up to a maximum of Q24W.

B) Visit Interval

Patients will visit trial sites every 4 weeks up to W24, and then every 8 weeks. In addition, patients will visit trial sites also at W52 and W60 for the trial assessments.

If active DME* is observed at W12 or subsequent visits, the patient will be transferred to the following regimen thereafter (Figure 1.1-1, Rescue Regimen).

A) Injection Interval

At a visit when active DME is observed, the patient will receive faricimab within the day. Then, patients will receive faricimab at an interval 4 weeks less than the time between the last dose and the visit date when active DME is observed. However, the minimum dosing interval should be Q8W. Thereafter, unless active DME is observed, the next and subsequent dosing intervals should be extended in a 4-week increments. The maximum dosing interval should be Q24W.

B) Visit Interval

Patients will visit trial sites every 4 weeks up to W24, and then every 8 weeks depending on a dosing interval. In addition, patients will visit trial also at W52, W56, W60, and W112 for the trial assessments.

* DME is regarded as active if CST is ≥ 325 μm** and clinically significant*** IRF or SRF is observed. Determination of disease activity will be performed for the study eye only.

** ≥ 325 μm with Spectralis SD-OCT and ≥ 315 μm with Cirrus or Topcon SD-OCT (or other equivalent OCT)

*** The condition is considered clinically significant if it is deemed to be a cause of vision loss or other aggravation of the disease.

A schematic of the trial schedule is presented below.

Figure 3.2-1 Schematic of the Trial Schedule

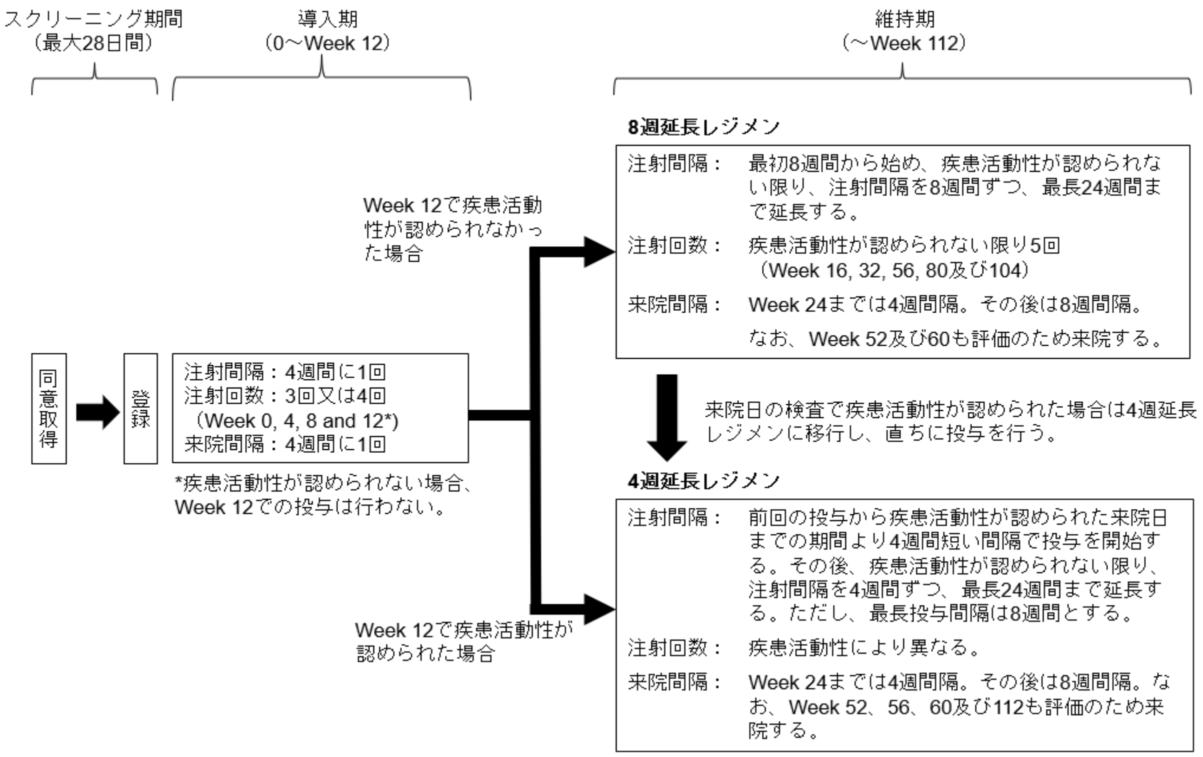


## 3.3 End of Trial and Length of Trial

The completion date of this clinical trial will be the date when the summary of the trial report is registered and published in jRCT.

The planned enrollment period and planned follow-up period are set as follows to allow for some cases of lost to follow-up and the time required for procedures for reporting to the CRB.

- Planned Enrollment Period: After a publication date on jRCT and 1 year from the first subject enrollment
- Planned Follow-up Period: After a publication date on jRCT and 2 years from the last subject enrollment

- Planned Report Preparation Period: 1 year from the end of the Follow-up period
- Planned Overall Trial Period: 4 years after a publication date on jRCT

### **3.4** Rationale for Trial Design

According to a nationwide survey on treatment with anti-VEGF drug administration for DME in Japan, 53.4% of patients received a single dose, 8.0% received two consecutive doses, 26.1% received three consecutive doses, and only 2.8% received five consecutive doses in the initial phase[13]. In the maintenance phase, the PRN regimen (administration only when the disease recurs) was predominant (75.0%), and the fixed-dose regimen and TAE regimens were less common. Problems in anti-VEGF therapy include the high economic and psychological burden on patients due to frequent administration[13].

In the phase 3 studies of faricimab, different dosing regimens were investigated; a regimen where faricimab 6.0 mg is administered Q4W 6 times during the initial phase, then Q8W during the maintenance phase, and a personalized treatment interval (PTI) regimen where faricimab 6.0 mg is administered 4 times Q4W during the initial phase, then at personalized intervals between Q4W and Q16W depending on patient's DME activity during the maintenance phase. Results of the Phase 3 studies showed that both dosing regimens provided visual acuity improvement comparable to that of the comparator drug (aflibercept). Most patients achieved CST < 325 μm by 12 weeks after the first administration, and more than half of the patients had received the study drug at a 16-week interval 1 year after the first administration in the PTI group[16]. These results indicate that a less frequent dosing regimen with reduced patient burden may maintain adequate efficacy, at least in some patients.

In this research, the 4$^{th}$ dosing in the initial phase can be skipped depending on the disease activity*, and dosing intervals from W12 onwards can be extended up to 24W. The regimen will be switched in case that active DME is observed: immediate dosing when active DME is observed, and the dosing intervals will be extended in a 4-week increments subsequently. As a criterion for disease activity, CST > 325 μm will be used, because it has also been used in clinical studies of faricimab and similar drugs. This new regimen is less burdensome for patients than the dosage regimen currently specified in the package insert, and is expected to improve adherence, leading to improvement of treatment outcomes.

* DME is regarded as active if CST is ≥ 325 μm** and clinically significant*** IRF or SRF is observed. Determination of disease activity will be performed for the study eye only.

** ≥ 325 μm with Spectralis SD-OCT and ≥ 315 μm with Cirrus or Topcon SD-OCT (or other equivalent OCT)

*** The condition is considered clinically significant if it is deemed to be a cause of vision loss or other aggravation of the disease.

### 3.5 Rationale for Sample Size

For the primary endpoint of change from baseline in BCVA at 1 year after treatment initiation, assuming a true value of 0.22 [logMAR] and a standard deviation of 0.22 [logMAR] based on the phase 3 clinical study results of faricimab (YOSEMITE and RHINE studies), 45 cases will provide > 80% probability that the point estimate of the primary endpoint is not more than 0.04 [logMAR] lower than the true value. In addition, assuming that the proportion of patients with a dosing interval of Q16W or more is 50% based on the clinical study results, the number of patients (23

cases) will provide > 80% probability that the point estimate is not more than 0.04 [logMAR] lower the true value in that population, and the 95% confidence interval is 4.48 letters. This is less than 0.09 [logMAR] (one ETDRS line), which is considered clinically significant. Dropout rates at 1 year in YOSEMITE and RHINE studies were approximately 10%. However, the sample size will be 70 assuming a 35% dropout rate in the first year since patients bear the drug cost and patient's burden of visit is heavy in this research.

### 3.6 Significance of Trial

The advent of anti-VEGF agents has greatly advanced the treatment of neovascular age-related macular degeneration and DME. On the other hand, frequent intravitreal administration of anti-VEGF agents imposes a heavy financial and psychological burden on patients, and in practice, treatment is often limited to the minimum necessary, with re-administration only when the disease recurs. However, inadequate treatment may lead to gradual progression of retinal damage, leading to irreversible visual impairment. If the efficacy and safety of the new dosing regimen of faricimab proposed in this trial are confirmed, it will provide a new treatment option with less patient burden, and is expected to fill the current clinical gap.

### 3.7 Benefits and Risks Involved in Trial Participation

Since all patients participating in this trial will receive treatment with faricimab for 2 years, improvement in DME is expected, although no benefits over normal clinical practice are expected. In addition, as academic achievement, the results of this trial may lead to future advances in the treatment of DME.

On the other hand, the number of visits and examinations may increase compared to usual medical care by participation in this trial, consequently resulting in an increase in the cost of examinations. In addition, there is a possibility that adverse events described in the Faricimab package insert may occur, as is the case when patients receive Faricimab in the usual medical practice. A list of adverse reactions confirmed to be related to faricimab is provided in Table 3.7-1 and a list of potential risks caused by examinations and procedures performed in this trial is provided in Table 3.7-2.

Table 3.7-1 Adverse Reactions of Faricimab

| **Important Adverse Reactions** | |
|---|---|
| Eye Disorders<br>• Intraocular inflammation (boudoir meningitis, nitritis, etc.) (1.0%)<br>• Retinal pigment epithelial lacunae (0.4%)<br>• Endophthalmitis (frequency unknown)<br>• Retinal exfoliation (frequency unknown)<br>• Omental laceration (frequency unknown) | |
| Stroke<br>• Ischemic stroke (0.05%)<br>• Thrombotic cerebral infarction (0.05%)<br>• Lacunar stroke (0.05%) | |
| **Other Adverse Reactions** | |
| Eye disorders (less than 1%) | Eye disorders (frequency unknown |

| | |
|---|---|
| • Increased intraocular pressure<br>• Vitreous floaters<br>• Ocular hypertension<br>• Corneal abrasion<br>• Eye pain<br>• Eye discomfort | • Conjunctival hemorrhage) |

Table 3.7-2 Possible Risks Caused by Examinations or Procedures

| Examinations/Procedures | Possible Risks |
|---|---|
| Intravitreal injection | • Bacterial infection from the injection site may occur. Antibacterial eye drops used to prevent infection may cause eye irritation, itching, swelling, or redness.<br>• Use of anticoagulants (e.g., aspirin or similar drugs [such as warfarin]) may predispose to intraocular bleeding associated with faricimab injections. |
| Blood test (approximately 10 mL blood is collected for HbA1c and serum creatinine measurement) | • Blood collection may cause pain, internal bleeding, or infection at the puncture site. Dizziness, fainting, or stomach upset may occur when blood is drawn. |
| Intraocular pressure measurement (when measuring by placing the intraocular pressure meter in contact with the cornea) | • Eye drops may be administered.<br>• Measurements will be taken at the time of medical examination and 30 minutes after faricimab injection. |
| Fluorescein angiography or indocyanine green angiography | • Before the imaging, fluorescein or indocyanine green will be injected into the arm. Puncture discomfort may occur.<br>• Dye injections can cause vein irritation and redness and swelling at the injection site.<br>• The common adverse reactions of fluorescein include nausea and vomiting, but sometimes allergic reactions, fainting, difficulty breathing, or shock may occur. Fluorescein may cause yellowing of the skin and urine, but this will disappear in about a day.<br>• The common adverse reactions of indocyanine green include nausea and vomiting, but sometimes allergic reactions and shock may occur. Indocyanine green may cause your stool to turn green, but this will clear up over time. |
| Use of povidone-iodine | • Povidone-iodine, which doctors administering faricimab injections use to prevent infection, may cause a brief burning, stinging, or irritation sensation in the eyes. |
| Use of mydriatics | • Mydriatics may cause eye irritation.<br>• After using mydriatics, blurred vision may occur for a while. Instruct patients not to drive or operate machinery until these |

| | |
|---|---|
| | symptoms resolve. |
| Use of antibacterial eye drops | • At investigators' discretion, antibiotic eye drops may be used before and after faricimab injections to prevent infection.<br>• Antibiotic eye drops may cause eye irritation, itching, swelling, and redness. |

For adverse events, measures to minimize the possible risk will be taken as described in Section 4.1.1 “Inclusion Criteria”, Section 4.1.2 “Exclusion Criteria”, and Section 5 “Treatment and Criteria for Treatment Change". In addition, a system to take necessary measures in case of serious or unexpected adverse events will be established.

# 4. Subjects and Methods

## 4.1 Subjects

### 4.1.1 Inclusion Criteria

Subjects who meet all of the following inclusion criteria will be enrolled in this trial.

**General Inclusion Criteria**

① Patients who provide written informed consent
② Age ≥ 18 at the time of consent
③ Confirmed diagnosis of diabetes mellitus (Type 1 or Type 2)
④ Ability and willingness to undertake all scheduled visits and assessments

**Inclusion Criteria for Study Eye**

One eye will be designated as a study eye. If both eyes are determined to be eligible, the eye with the worse BCVA at screening will be selected as a study eye, unless the investigator or co-investigator determines that the other eye is more appropriate for research treatment.

① Macular thickening secondary to DME involving the center of the fovea with CST ≥ 325 μm, as measured on Spectralis SD-OCT, or ≥ 315 μm, as measured on Cirrus SD-OCT or Topcon SD-OCT (or other equivalent OCTs) at screening
② BCVA of 0.0625 ~ 0.7 (decimal visual acuity) on visual acuity test at screening
③ Sufficiently clear optic media and adequate pupillary dilatation to allow acquisition of good quality color fundus photography (CFP) and other imaging modalities

### 4.1.2 Exclusion Criteria

Subjects who do not meet all of the following exclusion criteria will be enrolled in this trial.

**General Exclusion Criteria**

① History of severe allergic reaction or anaphylactic reaction to a biologic agent or known hypersensitivity to faricimab and any of its excipients, mydriatic eye drops, anesthetics or antimicrobials
② History of other disease, other non-diabetic metabolic dysfunction, physical examination finding, historical or current clinical laboratory finding giving reasonable suspicion of a condition that contraindicates the use of the faricimab or that might affect interpretation of the results of the trial or renders the patient at high risk for treatment complications in the opinion of the investigator or co-investigator
③ Active cancer within the past 12 months except for appropriately treated carcinoma in situ

of the cervix, non-melanoma skin carcinoma, and prostate cancer with a Gleason score of $\leq$ 6 and a stable prostate-specific antigen for > 12 months

④ Systemic treatment for suspected or active systemic infection

⑤ Participation in an investigational study that involves treatment with any drug or device (except for vitamins and minerals) within 3 months prior to Day 1

⑥ Administration of systemic pro-angiogenic treatments for the peripheral or coronary ischemia (e.g., limb ischemia or myocardial infarction) within 3 months prior to Day 1

⑦ Not willing to comply with the trial or follow-up procedures

⑧ Renal failure requiring renal transplant, hemodialysis, or peritoneal dialysis or anticipated to require hemodialysis or peritoneal dialysis at any time during the trial

⑨ Uncontrolled blood pressure (defined as systolic >180 mmHg and/or diastolic >100 mmHg while a patient is at rest). If a patient's initial reading exceeds these values, a second reading may be obtained later the same day or on another day during the screening period..

⑩ Stroke (cerebral vascular accident) or myocardial infarction within 6 months prior to Day 1

⑪ Pregnancy or breastfeeding, or intention to become pregnant during the trial

⑫ Women of childbearing potential* who does not agree to remain abstinent (refrain from heterosexual intercourse) or use acceptable contraceptive methods that result in a failure rate of < 1% per year** during the treatment period and for at least 38 days after the final dose of the IMP.

* Postmenarchall women who have not reached postmenopausal status (amenorrhea for at least 12 consecutive months with no cause other than menopause), and are not permanently infertile by surgery (removal of ovaries, fallopian tubes and/or uterus) or other causes as determined by the investigator or co-investigator (e.g., Müllerian duct dysplasia) considered women of child-bearing potential. According to this provision, women with unilateral tubal ligation are considered women of child-bearing potential.

** Examples of contraceptive methods with annual failure rates of less than 1% include bilateral tubal ligation, male sterilization, hormonal contraceptives that inhibit ovulation, hormone-releasing intrauterine devices, and copper-added intrauterine devices. The reliability of sexual abstinence should be evaluated with respect to the duration of the clinical research and each patient's preferences and normal lifestyle. Cyclic abstinence (calendar, ovulation day, symptomatic temperature, post-ovulation, etc.) and external ejaculation are not adequate contraceptive methods.

⑬ Requirement for continuous use of any prohibited concomitant medications or therapies listed in Section 5.4.4.

⑭ Patients who are not receiving treatment for diabetes.

**Exclusion Criteria for Study Eye**

① High-risk PDR in the study eye (using any of the following established criteria for high-risk PDR)

1) Any vitreous or pre-retinal hemorrhage
2) Neovascularization elsewhere $\geq$1/2 disc area within an area equivalent to the mydriatic ETDRS 7 fields on clinical examination or CFPs
3) Neovascularization at disc $\geq$1/3 disc area on clinical examination

② Tractional retinal detachment, pre-retinal fibrosis, vitreomacular traction syndrome, or epiretinal membrane involving the fovea or disrupting the macular architecture in the study eye
③ Active rubeosis
④ Uncontrolled glaucoma
⑤ History of retinal detachment or macular hole (Stage 3 or 4)
⑥ Aphakia or implantation of anterior chamber intraocular lens
⑦ Intravitreal administration of anti-VEGF agents within 3 months prior to Day 1 (applicable to patients whose study eyes were previously treated with intravitreal anti-VEGF agents), or any intravitreal administration of anti-VEGF agents to study eye prior to Day 1 (applicable for treatment-naive patients) Enrollment of patients who have a medication history of intravitreal administration of anti-VEGF agents should be no more than 25% of the total.
⑧ History of PRP, macular laser (focal, grid, or micropulse), any cataract surgery or treatment for complications of cataract surgery with steroids or YAG (yttrium-aluminum-garnet) laser capsulotomy within 3 months prior to Day 1
⑨ Any other intraocular surgery (e.g., corneal transplantation, glaucoma filtration, pars plana vitrectomy, corneal transplant, or radiotherapy)
⑩ Any intravitreal or periocular (subtenon) corticosteroid treatment within 6 months prior to Day 1
⑪ Treatment for other retinal diseases that can lead to macular edema
⑫ Any history of idiopathic or immune-mediated uveitis in either eye

**Exclusion Criteria for Non-study Eye**

① Non-functioning non-study eye, defined as either:
- BCVA of hand motion or worse
- No physical presence of non-study eye (i.e., monocular)

**Exclusion Criteria for Both Eyes**

① Prior intravitreal administration of faricimab in either eye
② Active ocular inflammation or suspected or active ocular or periocular infection in either eye on Day 1

**Exclusion Criteria for Concurrent Ocular Conditions**

① Any current or history of ocular disease other than DME that may confound the assessment of the macula or affect central vision in the study eye (choroidal neovascularization, age-related macular degeneration, retinal vein occlusion, uveitis, angioid streaks, histoplasmosis, active or inactive cytomegalovirus, pathological myopia, retinal detachment, retinal embolus, macular traction, macular hole, and other)
② Any current ocular condition that, in the opinion of the investigator or co-investigator, is currently causing or could be expected to contribute to irreversible vision loss due to a cause other than DME in the study eye (e.g., foveal atrophy, foveal fibrosis, pigment abnormalities, dense subfoveal hard exudates, or other non-retinal conditions)

## 4.2 Enrollment

### 4.2.1 Enrollment Procedure

In this trial, subjects will be enrolled via an Electronic Data Capture (EDC) system.

The investigator, co-investigators, and clinical research coordinators must be approved by a certified review board (CRB). After approval, they will obtain a user ID and password to log in to the EDC system from the Supportive Secretariat and register themselves.

The investigators or co-investigators will explain the trial to the prospective subjects. If written consent is obtained from the subject himself/herself, the investigators or co-investigators will access the EDC system via the Internet after confirming that the subject meets all the inclusion criteria and does not meet any of the exclusion criteria.

The enrollment is available 24 hours a day, except for the time of system check or maintenance for troubleshooting. The investigators and co-investigators will follow the instructions of the EDC system to enter the required fields and enroll a subject.

Name of EDC system: IBM Clinical Development
URL: https://www.ibm.com/jp-ja/products/clinical-development
Contact:
Company Name: IBM Japan Ltd.
Address: 19-21 Nihonbashi Hakozaki-cho, Chuo-ku, Tokyo 103-8510

### 4.2.2 Issue and Notice of Enrollment Result

Data entry into the electronic case report form (eCRF) will be available only for patients whose eligibility is confirmed based on the information entered on the registration screen.

### 4.2.3 Enrollment in Multiple Trials

Duplicate enrollment with this study is acceptable if the investigator or co-investigator determines that there is no problem with duplicate enrollment after reviewing the protocol for the other study. However, duplicate enrollment in this trial and other clinical research or study involving interventions is not allowed.

### 4.2.4 Precautions for Enrollment

Enrollment after the start of treatment is not allowed without exception. Enrollment is done by accessing the EDC system URL in "4.2.1 Enrollment Procedures". Eligibility is verified on the EDC system screen.

All data required at the time of enrollment is mandatory and must be authentic. If false enrollment is found after enrollment, it will be treated as a serious violation.

If input data is incomplete, enrollment will not be accepted until all data are entered.

Once enrolled, the subject’s enrollment will not be canceled (i.e., not deleted from the database) including the case of withdrawal of all consents including refusal to use the data for research purposes (however, in the case of withdrawal of all consents including refusal of research use of the data, the subject will be excluded from the analysis by data handling).

When an erroneous or duplicate enrollment is found, the investigator or co-investigator will notify the Supportive Secretariat as soon as possible. In the case of duplicate enrollment, information at the initial enrollment (enrollment number) will be used in all cases.

### 4.2.5 Procedure for Enrollment Closes

When the planned number of enrollments for this trial is expected to be achieved, the Supportive Secretariat will send an e-mail to the investigators informing them of the enrollment status. With regard to the end date of enrollment, the Supportive Secretariat will notify the trial sites that the planned enrollment number will be soon achieved and precautions for future subject enrollment (i.e., the site will not provide explanations to new subjects after the planned enrollment number is achieved).

## 4.3 Schedule of Activities

The schedule of observations and examinations for this trial is shown in Appendix 1. The investigator or co-investigator will conduct observations and examinations in accordance with the trial schedule. If all examinations are completed and evaluated on the same day or within 2 business days, the screening visit and the Day 1 visit may be performed together as a single visit. If the screening visit and Day 1 visit are performed on the same day, common examinations may be performed only once for both visits. No need to receive laboratory test results at screening before enrollment if such the past result can be utilized.

### 4.3.1 Informed Consent and Screening Log

Before a prospective subject participates in this trial, the investigator or co-investigator must fully explain this trial using an informed consent document and obtain written consent from the subject by his/her voluntary agreement. If a subject is unable to give written consent for any reason (in a case that a patient is unable to read a document due to visual impairment, etc. but is able to understand its contents through oral explanation, or a case that a patient is unable to sign a document due to limb impairment, etc. but is able to read and understand the contents of the document), a witness must be present for the explanation and consent. The witness must sign and date the consent document and certify that the prospective subject understands this study and has given his/her consent freely and voluntarily. The witness must not be a person engaged in this research. Consent documents of subjects will be kept at trial sites regardless of whether the subject is enrolled or not, and patients will receive a copy of the document.
The investigator or co-investigator will confirm the eligibility of a potential subject prior to enrollment. Results of tests and evaluations obtained in the routine medical practice prior to obtaining subject’s consent may be used, and there is no need to repeat the test or evaluation for screening purposes.

### 4.3.2 Medical and Surgical History, and Background Information

Medical history including clinically significant diseases, date of diagnosis of diabetes, visual acuity before getting DME, chronic and persistent complications, surgical history, and medication history all *prescribed drugs* taken prior to 7 days of Day 1) will be checked and recorded in the (eCRF during the screening period. An adverse event that occurs after consent is obtained and before the first dose of the IMP will be recorded in the eCRF as medical history or complication. For medical history, record Clinically significant illnesses that have been cured within the first 6 months of treatment with the drug will be recorded. For complications, any illnesses that have not been cured at the time of initiation of treatment with the drug will be recorded.
Gender, age, and race/ethnicity will be recorded in the eCRF as background information on the subject.

### 4.3.3 Body Weight and Height

Body weight and height will be measured during the screening period and recorded in the eCRF.

### 4.3.4 Vital Signs

Vital signs will be measured according to the Schedule of Activities (Appendix 1). Systolic and diastolic blood pressure, and pulse rate in the sitting position will be measured. Vital signs will be measured after 5 minutes of rest.

An abnormal finding observed before the first dose of IMP will be recorded in the eCRF as a complication. Any new clinically significant abnormal finding or worsening of a clinically significant abnormal finding observed at the subsequent visit should be recorded in the eCRF as an adverse event.

### 4.3.5 Ophthalmic Examination

The following ophthalmologic examinations will be performed according to the Schedule of Activities (Appendix 1). Unless otherwise specified, ophthalmic examinations should be performed on both eyes.

#### 4.3.5.1 Finger-counting Test

For a safety assessment, a finger-counting test of a study eye will be performed within 15 minutes after administration of IMP into the study eye to confirm that the patient has no visual problems. If visual impairment is observed, it should be recorded in the eCRF as an adverse event.

#### 4.3.5.2 Intraocular Pressure

Prior to eye dilation for ophthalmic examination, intraocular pressure in both eyes will be measured, and recorded in the eCRF. If intraocular pressure prior to eye dilation is greater than 30 mmHg, administration of a dilating drop and IMP should be discontinued. Intraocular pressure of the study eye will be measured also 30 minutes after administration of IMP whenever possible and recorded in the eCRF. If a sustained increase in intraocular pressure is observed, it should be recorded in the eCRF as an adverse event.

Intraocular pressure of a subject should be measured using the same method throughout the trial period.

#### 4.3.5.3 Refraction Test

A refraction test of both eyes will be performed. A refraction test should be performed prior to eye dilation. Spherical power, cylinder power, and cylinder axis at screening will be recorded in the eCRF. Any clinically significant anomalies of refraction (myopia, hyperopia, astigmatism, or heterophoria) should be recorded in the eCRF as a complication.

#### 4.3.5.4 Visual Acuity Test

A visual acuity test will be performed prior to eye dilation. The corrected visual acuity at a distance of 5 m will be measured using Landolt ring and decimal visual acuity will be recorded in the eCRF. The BCVA is expressed in logMAR converted from the decimal visual acuity using the following formula.

logMAR = log(1/d) (d = decimal visual acuity)

If the decimal visual acuity is less than 0.02, the visual acuity will be evaluated by finger counting, hand movement, or light perception.

#### 4.3.5.5 Slitlamp Microscopy

Slitlamp microscopy of both eyes will be performed after eye dilation to observe the anterior segment and optic media. Any abnormal findings should be recorded in the eCRF as complications or adverse events.

#### 4.3.5.6 Funduscopy

Indirect Ophthalmoscopy

Indirect ophthalmoscopy of both eyes will be performed after eye dilation to observe the fundus images. Any abnormal findings should be recorded in the eCRF as complications or adverse events.

Color Fundus Photography (CFP)

CFPs of both eyes will be taken under mydriasis according to the manual for fluorescence fundus angiography and color fundus photography. Image analysis of CFP (retinal hemorrhage, microaneurysm, soft or hard exudate, etc.) will be performed by the imaging CRO. The imaging procedure, transfer of CFP image data, image analysis, and reporting of analysis results will be described in the manual for fluorescence fundus angiography and color fundus photography.

Fluorescein Angiography (FA)

Fluorescence imaging of both eyes will be performed under mydriasis according to the manual for fluorescence fundus angiography and color fundus photography. The imaging procedure, transfer of FA image data, image analysis, and reporting of analysis results will be described in the manual for fluorescence fundus angiography and color fundus photography.

Indocyanine Green Angiography (ICGA)

If the investigator or co-investigator consideres it necessary, fluorescence ophthalmography of both eyes will be performed under mydriasis. After intravenous injection of 25 mg of indocyanine green at the elbow, imaging using an ultra-widefield scanning laser ophthalmoscope or other fundus camera will be performed. ICGA image data exchange and image analysis follow the manual for FA.

#### 4.3.5.7 Optical Coherence tomography (OCT)

Spectral-domain Optical Coherence tomography (SD-OCT)

SD-OCT scans of both eyes will be performed under mydriasis according to the manual for the OCT. The investigator or co-investigator will measure the CST from the tomogram of the macula, check for IRF and SRF, and record the results in the eCRF. At visits at W12 and thereafter, if the CST is greater than 325 μm and a clinically significant* IRF or SRF is observed, faricimab will be administered immediately (see section 5.2).

* IRF or SRF is considered clinically significant if it is deemed to be a cause of vision loss or other aggravation of the disease.

Other image analyses of SD-OCT will be performed by the imaging CRO. The imaging procedure, transfer of SD-OCT image data, image analysis, and reporting of analysis results will be described in the manual for the OCT.

OCT-Angiography (OCT-A)

At trial sites where OCT-A is available, OCT-A of both eyes will be performed to evaluate the vascular density and foveal avascular zone, etc. in the superficial capillary plexus and deep capillary plexus. Image analysis of OCT-A will be performed by the imaging CRO. Transfer of OCT-A image data, image analysis, and reporting of the analysis results will be described in a separate document.

### 4.3.6 ETDRS Diabetic Retinopathy Severity Scale (ETDRS DRSS)

The ETDRS DRSS is a rating scale that classifies the severity of DR according to its progression status[17]. The ETDRS DRSS is evaluated based on the results of CFP and FA. According to the Schedule of Assessments (Appendix 1), the personnel of the imaging CRO will evaluate the ETDRS DRSS for each of the study eye and non-study eye based on the following criteria.

Table 4.3-1 ETDRS-DRSS

| Level | ETDRS DRSS | Progression Status |
|---|---|---|
| 1 | 10, 12 | DR absent |
| 2 | 14A–14C, 14Z, 15, 20 | DR questionable, microaneurysms only |
| 3 | 35A–35F | Mild nonproliferative DR (NPDR) |
| 4 | 43A, 43B | Moderate NPDR |
| 5 | 47A–47D | Moderately severe NPDR |
| 6 | 53A–53E | Severe NPDR |
| 7 | 60, 61A, 61B | Mild proliferative PDR |
| 8 | 65A–65C | Moderate PDR |
| 9 | 71A–71D | High-risk PDR |
| 10 | 75 | High-risk PDR |
| 11 | 81 | Advanced PDR |
| 12 | 85 | Advanced PDR |
| 13 | 90 | Cannot grade |

### 4.3.7 Patient Reported Outcome

The following patient-reported outcomes will be collected using electric patient-reported outcome (ePRO). A dedicated application will be installed on the subject's own or rented smartphone or tablet device for use. Subjects will be instructed on the use of the ePRO prior to the start of the trial, and a user manual will be provided. If a subject has difficulty in responding to the questions due to a physical disability such as visual impairment, his/her family member may read out the questions and enter the answers on behalf of the subject.

#### 4.3.7.1 NEI VFQ-25

The NEI VFQ-25 is a rating scale to assess health-related quality of life (QOL) related to vision and consists of 25 questions (+ 6 additional questions) that comprise 12 subscales (General

health, General vision, Ocular pain, Near vision activities, Distance vision activities, Social functioning, Mental health, Role limitations, Dependency, Driving, Color vision, and Peripheral vision) [18]. Subjects will assess the NEI VFQ-25 according to the Schedule of Assessment (Appendix 1).

#### 4.3.7.2 WPAI

The WPI is a typical tool to assess work productivity outcomes and consists of 6 questions related to work productivity and impairment in activities[19]. Subjects and their family members will assess the WPI according to the Schedule of Assessment (Appendix 1).

#### 4.3.7.3 Survey of patient's subjective symptoms

Patients will record their subjective symptoms such as visibility daily when possible using the application for patients. The responses will be used for the following exploratory analyses at the Collaborative Institute, and the results will be summarized separately from a clinical research report of this study.

- Frequency of information input
- Exploration of internal and external factors for continuation/non-continuation of faricimab treatment
- Exploration of Unmet Medical Needs (UMN) in daily life in patients treated with faricimab
- Exploration of the frequency of input and its impact on patient satisfaction with treatment and willingness to continue treatment, etc.

#### 4.3.7.4 Patient's problems in daily life, questions, etc.

Patients will record their subjective symptoms such as visibility daily as much as possible, as well as any problems in daily life and questions about treatment at any time, in free-text or multiple-choice questions.

### 4.3.8 Clinical Laboratory Test

Blood will be collected at screening, W56, and W112 to measure HbA1c and serum creatinine level.

### 4.3.9 Unscheduled Visit

In the case that a patient needs to visit the trial site before the scheduled visit date (± visit window) (unscheduled visit), the tests to be performed on all other visits (vital signs, investigation of concomitant medications and therapies, adverse events, intraocular pressure, refraction test, visual acuity test, slit-lamp microscopy, fundoscopy and SD-OCT) should be performed, and other necessary tests will be performed at the discretion of the investigator or co-investigator.
If a patient is determined to have active DME* at an unscheduled visit, the following actions will be taken.

- If more than 8 weeks have elapsed since the last dose, administer the IMP and switch to the Extension-by-4W Regimen (the IMP may be administered even if the patient has already been in the Extension-by-4W Regimen).
- If no more than 8 weeks have elapsed since the last dose, the patient will be observed without dosing. If dosing is necessary, the patient will be dropped from the research.

* DME is regarded as active if CST is ≥ 325 μm** and clinically significant*** IRF or SRF is observed. Determination of disease activity will be performed for the study eye only.
** ≥ 325 μm with Spectralis SD-OCT and ≥ 315 μm with Cirrus or Topcon SD-OCT (or other equivalent OCT)
*** The condition is considered clinically significant if it is deemed to be a cause of vision loss or other aggravation of the disease.

#### 4.3.10 Patient Discontinuation from Trial

Subjects have the right to voluntarily withdraw from the trial at any time for any reason. In addition, the investigator and co-investigator have the right to withdraw a subject from the trial at any time. Reasons for withdrawal from the trial may include, but are not limited to, the following:

- Subject or his/her legal representative withdrawal of consent
- Any medical condition that the investigator or co-investigator determines may jeopardize the subject's safety if he/she continues in the trial
- Investigator or co-investigator determines it is in the best interest of the subject to discontinue the trial

If a patient discontinues participation in the research at his/her own will, the investigator, co-investigator, or research collaborator will record the date of discontinuation and the reason for discontinuation for each trial subject. As long as the trial subject cooperates, the investigations, observations, and examinations specified in this trial protocol will be conducted to ensure the safety of the trial subjects. It is not necessary to conduct all the tests at the time of discontinuation. Necessary tests will be conducted after confirming the subject's intention.
If the investigator or co-investigator determines that it is difficult to continue the research due to the occurrence of adverse events, etc., the research will be terminated, and the patient will be monitored thereafter. If the disease or infectious disease has not yet recovered at the time of termination or discontinuation of the research, observation will be continued as long as possible until the disease or infectious disease recovers or relieves.

#### 4.3.11 Site Discontinuation

If the investigator discontinues or suspends the trial, the investigator will promptly inform the administrator of the trial site of the discontinuation/suspension and provide a detailed explanation in writing. The administrator of the trial site will promptly inform the Collaborative Institute and the Principal Investigator in writing of the discontinuation/suspension and provide a detailed explanation in writing.
The Collaborative Institute and the Principal Investigator will report discontinuation of the trial site from the trial at the continuing review by CRB, and change the information registered in the database maintained by the Ministry of Health, Labour and Welfare (jRCT*).

* Database maintained by the Ministry of Health, Labour and Welfare as stipulated in Article 24, Paragraph 1 of the Ordinance for Enforcement of Clinical Trials Act (Japan Registry of Clinical Trials: Publication System of Clinical Trial Plans/Reports)
URL: https://jrct.niph.go.jp/

#### 4.3.12 Discontinuation of the Entire Research

If any of the following criteria are met, the Investigator will consider discontinuing or suspending this entire clinical research, and will promptly notify each investigator of the decision to discontinue or suspend, together with the reasons for the decision. The Investigator will also notify the certified review board (CRB) specified in the trial protocol, of the discontinuation or suspension of this entire research within 10 days from the date of discontinuation. Notification will be made to the Minister of Health, Labour and Welfare using the Form 4. If necessary, the investigator will ask the certified review board for its opinion on the timing and method of termination of the research in accordance with the measures to be taken by the subject. Even after the notification of discontinuation is submitted to the Minister of Health, Labour and Welfare, reports on diseases, etc., and periodic reports, etc., should be made until the study is terminated (when all treatments for subjects are completed and the study is terminated). The investigator will promptly notify the administrator of the institution where the research is being conducted and the participating trial subjects, and will conduct examinations and appropriate measures to confirm the safety of the trial subjects.

Discontinuation Criteria

1) If a fact or information that undermines or may undermine the ethical validity or scientific rationality of this clinical research and is considered to affect the continuation of this clinical research is obtained.
2) If a fact or information that undermines or may undermine the appropriateness of the conduct of this clinical research or the reliability of the results of this clinical research is obtained.
3) If it is determined that the expected risks are higher than the expected benefits from this clinical research, or if information suggesting that adequate results have been obtained or that adequate results will not be obtained from this clinical research is obtained.
4) If important information regarding the quality, safety, or efficacy of the research drug is obtained, and it is determined that this clinical research as a whole cannot continue due to such information.

If it is found that a trial site (including persons engaged in the research) has committed a serious violation of the Clinical Research Act, this clinical trial protocol, or the agreement, or if it becomes impossible to properly conduct this clinical research, the trial site may be requested by the Investigator to discontinue or suspend the research. In addition, in the event of discontinuation or suspension due to any of the following reasons, arising at the trial site, the Investigator will promptly notify the participating trial subjects of such discontinuation or suspension, and will conduct tests to confirm the safety of the trial subjects and take appropriate measures.

1) When the CRB recommends or directs discontinuation
2) When the CRB instructs to change the research plan, etc., and it is deemed difficult to accept the change.

## 5. Treatment and Criteria for Treatment Change

### 5.1 Investigational Medicinal Product (IMP)

Generic Name: Faricimab (genetical recombination)

Brand Name: VABYSMO® solution for intravitreal injection

Route of Administration: Intravitreal

Dosage and Administration specified in the label (for DME):

Faricimab (genetic recombination) 6 mg (0.05 mL) is administered intravitreally every 4 weeks for usually 4 doses but fewer times depending on patient's symptoms. Then the dosing interval may be gradually extended and faricimab is usually administered intravitreally every 16 weeks. The dosing interval may be modified depending on patient's symptoms but 4 weeks at the shortest.

Marketing Authorization Holder: Chugai Pharmaceuticals Co., Ltd.

This research will be conducted using pharmaceutical products. manufactured after taking necessary measures to ensure the quality of the products to be used in this research.
In addition, records will be prepared or obtained concerning the following items related to the pharmaceuticals to be used in this research.

① The date of manufacture, manufacturing number or code of the drug product used in this research and other records related to the manufacture of the drug product
② In cases where the pharmaceutical products used in this research have been obtained, the quantity and date of delivery.
③ Records of the disposition of the drug products used in this research

5.2 Treatment

**Extension-by-8W Regimen**

Subjects participating in the trial will receive faricimab 6.0 mg intravitreally for 2 years. During the initial phase, all subjects will receive faricimab Q4W for 4 consecutive doses. However, the 4$^{th}$ dosing may be skipped depending on the disease activity. The dosing schedule from W12 onwards will be determined based on DME activity* at W12 and afterward. For subjects with no DME activity, the dosing interval from W12 onwards will begin at Q8W and be extended every 8 weeks thereafter (Q8W, Q16W, and then Q24W). The maximum dosing interval is Q24W.
For intravitreal administration of faricimab, be sure to check the latest package insert[8] and practical guide for appropriate use[20].

**Extension-by-4W Regimen**

If active DME* is observed at W12 or any subsequent visits, the treatment will be immediately switched to the Extension-by-4W regimen. In the Extension-by-4W regimen, a patient will receive faricimab immediately at the visit when active DME* is observed. Next, faricimab will be administered at intervals of 4 weeks shorter than the time between the last dose and the visit when active DME is observed. However, the minimum interval should be 8 weeks. Afterward, the subsequent dosing intervals will be extended by 4-week intervals unless active DME is observed. The maximum dosing interval is 24 weeks.

* DME is regarded as active if CST is ≥ 325 μm** and clinically significant*** IRF or SRF is observed. Determination of disease activity will be performed for the study eye only.

** ≥ 325 μm with Spectralis SD-OCT and ≥ 315 μm with Cirrus or Topcon SD-OCT (or other equivalent OCT)

*** The condition is considered clinically significant if it is deemed to be a cause of vision loss or other aggravation of the disease.

Even if CST < 325 μm, the investigator or co-investigator should consider the need to administer faricimab if a significant visual acuity loss since the previous visit is observed (e.g., decrease from 0.6 to 0.3 in decimal visual acuity), and if necessary, switch to the Extension-by-4W regimen.

## 5.3 Criteria for Treatment Interruption/Discontinuation

The trial treatment should be suspended or discontinued if any of the following criteria are met. The investigator or co-investigator will record the reason for interruption/discontinuation of the trial treatment on the appropriate eCRF page, and on the Adverse Events page of the eCRF in the case of interruption/discontinuation due to an adverse event.

Table 5.3-1 Criteria for Treatment Interruption/Discontinuation

| Event | Criteria |
|---|---|
| Intraocular inflammation | • Interrupt the trial treatment if intraocular inflammation (iritis, iridocyclitis, or vitritis) is $\geq$2+ in the study eye.<br>• The trial treatment may be resumed subsequently as determined by the investigator or co-investigator. |
| Cataract surgery in the study eye | • Interrupt the trial treatment after cataract surgery in the study eye.<br>• The trial treatment may be resumed no earlier than 28 days after uncomplicated cataract surgery and no evidence of post-operational inflammation at that time. For cataract surgery with complications, the trial treatment may be permitted as determined by the investigator or co-investigator. |
| BCVA decrease | • Interrupt the trial treatment if there is a treatment-related decrease in BCVA of > 0.6 logMAR in the study eye compared with the last assessment of BCVA prior to the most recent treatment.<br>• The trial treatment may be permitted subsequently as determined by the investigator or co-investigator. |
| Elevated Intraocular pressure | • Interrupt the trial treatment if pre-treatment intraocular pressure in the study eye is $\geq$30 mmHg.<br>• The trial treatment may be permitted when intraocular pressure has been lowered to <30 mmHg, either spontaneously or by treatment, as determined by the investigator or co-investigator. |
| Rhegmatogenous retinal break | • Interrupt the trial treatment if a retinal break is present in the study eye.<br>• The trial treatment may be resumed no earlier than 28 days after successful laser retinopexy, as determined by the investigator or co-investigator. |
| Rhegmatogenous retinal detachment or macular hole | • Interrupt the trial treatment if rhegmatogenous retinal detachment or Stage 3 or 4 macular hole occurs in the study eye.<br>• The trial treatment may be permitted subsequently as determined by the investigator or co-investigator. |
| Active infection or suspected active | • Interrupt the trial treatment if an active infection (e.g., infectious conjunctivitis, infectious keratitis, infectious scleritis, |

| | |
|---|---|
| infection | endophthalmitis) is observed or suspected, or if the patient requires treatment for an active systemic infection.<br>• Administration of the research drug may be allowed at the discretion of the investigator or co-investigator. |
| Use of prohibited concomitant drugs or therapies | • The trial treatment may be discontinued as determined by the investigator or co-investigator if a patient uses the prohibited concomitant drug/therapy listed in Section 5.4.4. |

If dosing is interrupted for any reason and then resumed, the following procedure should be followed.

- The research drug will be administered at the time of resumption, and the subsequent dosing schedule should be determined according to disease activity at the time of resumption.

## 5.4 Concomitant and Supportive Therapy

### 5.4.1 Prescribed Concomitant and Supportive Therapy

Not applicable

### 5.4.2 Recommended/Unrecommended Concomitant and Supportive Therapy

Not applicable

### 5.4.3 Permissible Concomitant and Supportive Therapy

Some common therapies that are allowed to be used concomitantly with faricimab are listed below.

- Onset of ocular hypertension or glaucoma in the study eye during a subject’s trial participation should be treated as clinically indicated.
- Onset of cataract or posterior capsular opacification in either eye during a subject’s trial participation may be treated as clinically indicated. Dose interruption criteria (see Section 5.3, Table 5.3-1) will be applied for cataract surgery.
- Short-term use of topical ocular corticosteroids after cataract surgery, YAG capsulotomy, peripheral iridotomy, argon/selective laser trabeculoplasty, or ocular allergic conditions.
- PRP may be allowed for the treatment of DR if the investigator or co-investigator judges it is necessary.
- Non-study eye treatment with anti-VEGF therapy (At the discretion of the investigator or co-investigator, patients may have their non-study eye treated with anti-VEGF therapy licensed for ocular use. However, administration into both eyes on the same day at the initial treatment should be avoided, and the contralateral eye should be treated after thorough safety assessment in one eye.)

### 5.4.4 Prohibited Concomitant Drug and Therapy

The following medications and treatments are prohibited from use during subject’s participation in the trial. Subjects will be discontinued from the trial when using these medications or treatments.

- Systemic anti-VEGF therapy
- Systemic drugs known to cause macular edema (fingolimod, tamoxifen)
- Intravitreal administration of anti-VEGF agents (other than faricimab) into study eye

- Intravitreal, periocular (subtenon), steroid implants, or chronic topical ocular corticosteroids into study eye
- Photodynamic therapy (PDT) to study eye
- Micro-pulse or grid pattern photocoagulation
- Vitreous surgery or pan-retinal photocoagulation in study eye
- Kallidinogenase (for improvement of symptoms of circulatory disturbance of the retinal choroid)
- Other experimental therapies (except those comprising vitamins and minerals)

# 6. Safety Assessments

## 6.1 Expected Adverse Events of Individual Drugs

For detailed information on individual drugs used in this trial, please refer to the most recent package insert. (Reference: Pharmaceuticals and Medical Devices Agency https://www.pmda.go.jp/PmdaSearch/iyakuSearch/)

## 6.2 Definition of Adverse Event

### 6.2.1 Definition of Adverse Event

An adverse event is defined as any unfavorable and unintended illness or sign (including an abnormal laboratory finding) occurring in a subject, regardless of causal relationship to the conduct of the trial. However, symptoms that occur with the progression of the underlying disease, which is the reason for the use of the IMP in this trial, shall not be handled as adverse events. All adverse events occurring after the first dose of the IMP and until the last visit day are subject to evaluation in this trial. An adverse event will be followed until it resolves, is resolving, or stabilizes.

### 6.2.2 Definition of Adverse Reaction

Among adverse events, disease, disability, death, infection, abnormal laboratory values, and various symptom suspected of being due to the conduct of the trial, are considered “disease or like”. In this trial, if there is a reasonable possibility between an adverse event and the IMP or performance of the tests specified in the trial protocol, such an event is considered as “disease or like”.
The term "infectious disease" means the case in which a biological product is suspected of containing a pathogen from a biologically-derived raw material or material into the relevant pharmaceutical product, etc. In addition, positivity of viral markers such as HBV, HCV, and HIV is also subject to reporting of infectious diseases.

### 6.2.3 Other Adverse Events

Since the information collected in ePRO may include the safety information that needs to be reported and handled in accordance with the regulations, patients will be instructed to inform the Investigator or co-investigator of information on health status and changes in physical condition.

### 6.2.4 Seriousness of Adverse Event

For each adverse event, the seriousness of the events will be judged on a two-level scale of 0. non-serious and 1. serious.

In this trial, the following case will be handled as a serious adverse event in accordance with the Pharmaceutical Affairs Law.

<Definition of a serious adverse event>

1) Is fatal
2) Is life-threatening
3) Requires or prolongs inpatient hospitalization for treatment
4) Results in persistent or significant disability/incapacity
5) Is a congenital anomaly/birth defect in a neonate/infant
6) Is an event or response considered to be a medically significant condition

### 6.2.5 Severity of Adverse Event

Criteria for determining the severity of adverse events are listed below.

Table 6.2-1 Classification Scale for Severity of Adverse Event

| Severity | Definition |
|---|---|
| Mild | Discomfort noticed, but no disruption of normal daily activity |
| Moderate | Discomfort sufficient to reduce or affect normal daily activity |
| Severe | Incapacitating with inability to work or to perform normal daily activity |

Note: Regardless of severity, some events may also meet seriousness criteria. Refer to the definition of a serious adverse event (see Section 6.2.4)

### 6.2.6 Criteria for Determination of Causal Relationship

Criteria for determining the causal relationship of an adverse event are as follows.

Table 6.2-2 Criteria for Determination of Causal Relationship between Adverse Event and Treatment

| Relationship | Criteria |
|---|---|
| Yes | There is a reasonable possibility that the adverse event is related to faricimab.<br>• The event reappears upon re-administration.<br>• The event can be expected based on the mechanism of the drug / The causal relationship has already been established including other drugs in the same drug class.<br>• There is a temporal relationship between faricimab and the onset of the event.<br>• The event disappears after discontinuation of faricimab only.<br>• The event cannot be explained by an underlying disease, complication, concomitant therapy, etc. |
| No | A relationship between the adverse event and faricimab can be ruled out.<br>If the case meets any of the following.<br>• There is no temporal relationship between faricimab and the onset of the event.<br>• The event can be reasonably explained by any other factor (e.g., underlying |

| disease, complication, medical history, concomitant therapy, etc.) |
|---|

# 7. Adverse Event Reporting

## 7.1 Adverse Event Reporting Period

After the first dose of faricimab in this trial, all adverse events that occur up to the date of the last visit, or the date of withdrawal of consent or lost to follow-up are subject to reporting regardless of relationship to the drug. The investigator or co-investigator will record the following items in the eCRF.

- Subject ID
- Sex
- Age
- Dosing Period (Start date, end date, duration, dosage)
- Adverse event name
- Onset
- Outcome
- Outcome confirmation date
- Causal relationship (Investigator's or co-investigator's opinion)
- Lot number

* In the case of a serious adverse event, the course of the event and the investigator's comment should be added to the items above.

## 7.2 Actions When Any Adverse Event Occurs

The investigator or co-investigator will make effort to collect information on adverse events when he/she has contact with a subject, and will update the information in the eCRF. All adverse events should be recorded in the subject's medical record, regardless of whether it is reported by a subject or found by trial personnel at a trial site. When the Supportive Secretariat obtains information on an adverse event from a trial site, they will report it to Chugai Pharmaceutical Co. Ltd. based on a contract.

Chugai Pharmaceutical Co., Ltd. will evaluate information on an adverse event obtained, and report the information to the Pharmaceuticals and Medical Devices Agency or companies having a contract concerning the transfer of the safety information, as necessary. In addition, Chugai Pharmaceutical Co., Ltd. will take measures such as informing trial sites of the matters to be disseminated as necessary. The investigator or co-investigator will cooperate with additional investigations regarding inquiries from Chugai Pharmaceutical Co. Ltd. whenever possible.

The Principal Investigator will make a decision on adverse events obtained from trial sites, considering the urgency, importance, and impact of the reported matter, and seeking the opinion of the Research Executive Committee members as necessary.

## 7.3 Actions in Case of "Disease or Like" or Infection

When the investigator and co-investigator obtain information on "disease or like" or infection, they will report such information following the procedures 1) through 6) below.

When the co-investigator obtains the information, he or she should promptly inform the investigator and take the same action, however, if the investigator cannot be contacted, the co-investigator must act on behalf of the investigator.

1) When the investigator obtains information on serious “disease or like”, serious infections, or non-serious unexpected infections among adverse events, the investigator will promptly report the information to the administrator of the trial site, fill in the specified items on the Uniform Form 8 “Report of Disease or Like” (hereinafter referred to as "Form 8"*) to the extent possible, and send it to the Principal Investigator, the Research Secretariat, and a person in charge of pharmacovigilance in the Supportive Secretariat within 10 days by e-mail. If new information is obtained after the initial report, the information should be added to the Form 8 and reported promptly to the Principal Investigator/ Research Secretariat/ Supportive Secretariat.
2) After obtaining the Form 8, the Principal Investigator/Research Secretariat/Supportive Secretariat will submit the Form 8 to the Research Secretariat in accordance with the reporting deadlines specified below. With this reporting, it shall be deemed that the event was reported to the administrator of the trial site to which the Principal Investigator belongs and to the CRB. However, in urgent cases, such as when information on an unexpected death is obtained, the report should be promptly made to the administrator of the trial site to which the Principal Investigator belongs. The method of reporting may be by telephone or orally.

| | Other | | Infection | |
|---|---|---|---|---|
| | Unexpected[a] | Expected[b] | Unexpected[a] | Expected[b] |
| **Death** | 15 days | 15 days | 15 days | 15 days |
| **Serious** | 15 days | 30 days | 15 days | 15 days |
| **Non-serious** | periodical report | periodical report | 15 days | periodical report |

a) The Investigator or co-investigator should handle the disease, etc. as "unexpected" if the occurrence of the disease, etc. is not described in any of the following documents and cannot be predicted: (1) trial protocol or informed consent documents, (2) important adverse reactions and other adverse reactions in the package insert of the drug used in this research, and (3) Guide for the proper use of drugs to be used in this research.

b) If the disease, etc. that has occurred is described in documents (1), (2) or (3) above, it should be handled as expected.

3) After reporting to the Research Secretariat in 2), the Principal Investigator/Research Secretariat/Supportive Secretariat will inform the investigators of all trial sites that they had reported the event to the CRB, and provide the information to the investigators. In addition, a copy of the submitted "Form 8" will be provided to the Collaborative Institute without delay after reporting to the CRB.
4) The investigators of all trial sites will report the content of the information to the administrator of each trial site to which they belong.
5) When the Principal Investigator/ Research Secretariat/ Supportive Secretariat obtain the review results of the information from the CRB, they will report it to the Director of Shinshu University Hospital and also report the review results to the investigators of all trial sites.
6) The investigators of all trial sites will report the review results from the CRB to the administrator of each trial site to which they belong.

* The latest version of "Form 8" should be used. It is available on the MHLW website.
http://www.mhlw.go.jp/stf/seisakunitsuite/bunya/0000163417.html

When the CRB receives a report of disease, etc., and deems it necessary, the CRB will express its opinion to the investigator or principal investigator on measures to be taken to investigate the cause of the disease, etc. or to prevent recurrence of the disease, etc. pertaining to the report. When the CRB receives a report on a disease, etc., and expresses its opinion to the investigator or principal investigator, the investigator or principal investigator must respect the opinion and take necessary measures.

### 7.4 Follow-up of Patients after Adverse Event

If an adverse event (including adverse events related to laboratory test abnormality) is observed after the first dose of faricimab in this trial, the investigator or co-investigator will take the best possible measures and treatment. If the investigator or co-investigator judges that continuation of the trial is difficult due to the occurrence of adverse events, the trial will be terminated, and the patient will be monitored thereafter.
If “disease or like” or infection has not resolved at the time of completion or discontinuation of the trial, it should be followed as long as possible until it resolves or mostly resolves.
If an outcome of the adverse event changes while the patient is in the trial, it should be recorded in the source documents and eCRF, along with the date.

### 7.5 Pregnancy in Female Patients

Female subjects will be instructed with the consent document to immediately report the pregnancy to the investigator or co-investigator if she is confirmed to be pregnant during the trial or within 38 days after the last administration of faricimab. When the investigator or co-investigator knows the fact of pregnancy, he or she will report it to the Research Secretariat via the " Investigation Report Form on Pregnancy Case" (Annex 3) and take appropriate action. Pregnancies of female subjects will not be recorded in the eCRF.
The health status of a birth will be recorded on the "Investigation Report on Pregnancy Cases" (Appendix 3) and submitted to the Research Secretariat. In addition, information on the health status of a newborn at 6 and 12 months after birth will be recorded on the "Survey Report on the Newborns" (Appendix 4) and submitted to the Research Secretariat.

### 7.6 Reporting of Overdose/Dosing Error

If an event corresponding to an overdose or medication error with faricimab is identified in the opinion of the investigator or co-investigator, he or she will report it to the Research Secretariat via the "Investigation Report Form on Overdose/Medication Error" (Annex 2).

## 8. Statistical Considerations and Analysis Plan

Details of the statistical analysis will be described in a separate statistical analysis plan to be prepared prior to database lock.

### 8.1 Planned Number of Patients and Estimated Trial Period

The planned number of subjects is determined at 70 patients with DME based on the rationale described in "Section 3.5 Rationale for Sample Size”.
The estimated enrollment period and follow-up periods are determined as follows to allow for a small number of lost to follow-up and the time required for procedures for the CRB.

- Planned enrollment number: 70 patients with DME

- Estimated enrollment period: After a publication date on jRCT and 1 year since enrollment of the first patient
- Estimated follow-up period: After a publication date on jRCT and 2 years since enrollment of the last patient
- Period for preparation of a clinical trial report: 1 year from the end of the follow-up period
- Estimated entire trial period: 4 years after a publication date on jRCT

## 8.2 Analysis Populations

Analytical populations are defined as follows.

- Full Analysis Set (FAS): The FAS is defined as all patients who receive at least one dose of faricimab in this trial and have valid post-dose efficacy data.
- Per-protocol Set (PPS): The PPS is defined as all patients in the FAS who do not have a major protocol violation.
- Safety Analysis Set: The Safety Analysis Set is defined as all patients who receive at least one dose of faricimab in this trial.

## 8.3 Analysis of Primary Endpoint

The primary efficacy endpoint of this trial is the change from baseline in BCVA at 1 year after treatment initiation. The BCVA is expressed in logMAR values converted from decimal visual acuity measured at a distance of 5 m using a Landolt ring.

logMAR = log(1/d) (d = decimal visual acuity)

If the decimal visual acuity is less than 0.02, the visual acuity will be evaluated by finger counting, hand movement, or light perception. In such cases, the decimal visual acuity and logMAR values will be converted in accordance with the following table.

| | Decimal Visual Acuity | logMAR |
|---|---|---|
| Finger Counting (50 cm) | 0.01 | 2.0 |
| Hand Movement | 0.005 | 2.3 |
| Light Perception | 0.002 | 2.7 |
| Loss of Light Perception | 0.001 | 3.0 |

The Mixed Effect Model for Repeated Measure (MMRM) will be used for analyses of the BCVA. The model will include visit (categorical variable) and baseline BCVA (continuous variable) as fixed effects, and assume an unstructured covariance structure for modeling of intra-subject error. For the data missing mechanism, missing-at-random (MAR) will be assumed. The average values of W52, W56, and W60 will be calculated.
As the secondary analysis, the approximate numbers of ETDRS letters converted from logMAR using the following formula [21] will be analyzed in the same way as for the primary endpoint.

Number of ETDRS letters = 85 – 50 x logMAR = 85 – 50 x log(1/d) (d = decimal visual acuity)

If the decimal visual acuity is less than 0.02 (evaluated by finger counting, hand movement, or light perception), the number of ETDRS letters is calculated as zero.

### 8.4 Analysis of Secondary Endpoints

Details of the analysis plan for the secondary endpoints will be described in the statistical analysis plan.

### 8.5 Analysis of Exploratory Endpoints

Details of the analysis plan for the exploratory endpoints will be described in the statistical analysis plan.

### 8.6 Interim Analysis

Some analyses including the primary analysis will be performed when BCVA data at 1 year after treatment initiation are available for all patients.

### 8.7 End of Trial

The Principal Investigator will prepare a summary report of the primary endpoint , a clinical trial report, and a synopsis of the clinical trial report. After hearing the opinion of the CRB, the Principal Investigator will report to the parties involved in the trial that the trial is completed, and report to the administrator of the study site to which the principal Investigator belongs in writing on the completion of the trial and submit a synopsis of the clinical trial report. Each report will be submitted to the Minister of Health, Labour and Welfare and published in the jRCT maintained by the MHLW.
The completion date of this clinical trial shall be the date when a summary of the clinical trial report is registered and published in the jRCT.

## 9. Collection and Management

### 9.1 Data Quality Assurance

A data management person of the Supportive Secretariat will be responsible for data management of this trial, including quality check of data. The data to be collected via EDC by entering the data in eCRF. Trial sites will be responsible for data entry into EDC. If any discrepancy in the data is identified, the data management person will request the trial site to correct the data, and the trial site will resolve the data discrepancies electronically in the EDC. The eCRF and its change logs will be maintained in the EDC as an audit trail. System backup in the Supportive Secretariat and storage of trial data will follow the SOPs in the Supportive Secretariat.

### 9.2 Electronic Case Report Form (eCRF)

The investigator or co-investigator will prepare and submit the CRFs for all enrolled patients.
The entry into the eCRF will be made using the EDC system described in Section 4.2 Enrollment. Accounts in the eCRF will be issued to the investigators, co-investigators, and clinical research coordinators at each trial site. The eCRF will be electronically transmitted to and stored in the database and handled in accordance with a separately defined document.
The entry into the eCRF will be performed by persons in charge at the trial site who have the account. The investigator or co-investigator will review the contents of the eCRF and sign it electronically.

### 9.2.1 Input Items of eCRF

See Appendix 2.

## 9.3 Identification of Source Data

### 9.3.1 Data Recorded Only in eCRF Only

For the following items that are entered only in the eCRF, the eCRF shall be used as the source data.

1) Comments by the investigator or co-investigator
2) Dosing status: Dosing interruption or discontinuation, reasons for a change in dosing schedule
3) Adverse events: Severity of adverse events, seriousness, causal relationship, outcome, and date of confirming the outcome
4) Concomitant drugs/therapies: Purpose of use of concomitant drugs or therapies
5) Discontinuation of trial: Reasons for discontinuation

### 9.3.2 Source Document

Source documents are the materials (paper or electronic) in which the subject data were recorded. Source documents include, but are not limited to the following, inpatient and outpatient records, medical records, clinical laboratory test results, memos, drug prescription records, recorded data from automated analytical devices, photocopies that have been verified for accuracy and integrity, microfiche, photographic negatives, microfilm or magnetic media, radiographs, subject files, and various records kept in the pharmaceutical department, clinical laboratories, and medical technology departments involved in the clinical trial.
Source documents needed for verification of the validity and integrity of data entered into the eCRF must not be deleted or destroyed and must be retained in accordance with the policy on record retention.
Data entered in the ePRO will be retained on the vendor's server as source documents.
The (principal) investigators and the sites will make all source documents and other relevant clinical records available for source document verification during monitoring (audit) and investigations by the certified review board and regulatory authorities in connection with this research.

### 9.3.3 Use of Computerized Systems

If the clinical findings are entered directly into an electronic medical record system of a trial site as a substitute for the original hard copy record, the electronic record can be regarded as the source data.
The computerized data collection system used in the trial must store the data that was originally entered (original data). In other words, when the original data is changed, there must be an audit trail on the system that allows confirmation of the original data, the reason for the change, the person who made the change, and the date and time of the change.

## 9.4 Handling and Storage of Record, Information, and Sample

### 9.4.1 Trial site

The study sites will make records with regards to the following items for each. When these records and case report forms are changed or amended, the name of the person who made the

amendment, the date and reason of the amendment, and the history of the amendment should be recorded and retained with the amended records.

1) Date and time when, and place where the drug was used. (Each subject's medical record that describes when the drug was used in the study site is acceptable. In the case of an outpatient visit, it should be described state that it was conducted as an outpatient visit in accordance with the content of this study).
2) Matters that identify the subjects of this research.
3) Matters related to the medical treatment and examination of the subject of this research (records obtained from the subject through the implementation of this research for the evaluation items pre-specified in the trial protocol).
4) Matters related to participation in this research.
5) Other matters necessary for conducting this research.

In addition, the following documents, along with the above records, should be kept for 10 years after the publication (such as research papers) of the results of the research.

1) Documents or copies of trial protocols, implementation plan, documents pertaining to the explanation to the subjects of this research and their consent, summary reports, and other documents prepared by the (coordinating) investigator pursuant to the provisions of the Ordinance for Enforcement Regulations of the Clinical Research Act.
2) Documents received from a certified review board pertaining to the review opinion work.
3) Documents related to monitoring and auditing (limited to cases where such monitoring and auditing are conducted)
4) Source documents, etc.
5) Contracts related to the implementation of this research.
6) Documents describing the outline of pharmaceuticals, etc. to be used in this research.
7) Other documents necessary for conducting this research.

The investigators will retain important documents related to the implementation of the research (copies of applications, notification documents from the hospital director, copies of various applications and reports, consent forms, and other documents or records necessary to ensure the reliability of data) for 10 years after the publication of the results in a research paper or other publication. After that, they will be disposed of with care for personal information.
Use of data after withdrawal of consent will be confirmed with the subject at the time of withdrawal of consent. If, as a result of the confirmation, it is not possible to use the data until the time of withdrawal of consent, the data will be destroyed by the end of the study.
Residual biological specimens from laboratory tests will be disposed of appropriately in accordance with the rules of each trial site.

#### 9.4.2 Collaborative Institute

The Collaborative Institute will preserve documents or records with regard to the trial that should be preserved by the Collaborative Institute in accordance with the SOPs prepared and maintained by Chugai Pharmaceutical Co. Ltd. concerning retention, storage and disposition of record and information of a clinical research, The retention period will be until the later date of either 5 years after termination or completion of the entire trial or 3 years after the final report of the trial results. However, the data set (eCRF and summary data from the other information

source) will be stored until the later date of either 5 years after termination or completion of the entire trial or 25 years after the final report of the trial results. After the retention period, the data sets will be properly disposed of in accordance with the SOPs prepared and managed by Chugai Pharmaceutical Co. Ltd. In this research, the collaborative institute will not handle or store blood or other biological samples. The handling of data after withdrawal of consent is described in Section 10.2.2.

### 9.4.3 Supportive Secretariat

Information entered in the eCRF during this trial will be stored on the server of the Supportive Secretariat, which manages the EDC system. The data entered in the EDC system will be locked and then analyzed.

The Supportive Secretariat will appropriately store documents or records related to this trial in accordance with the SOPs of the Supportive Secretariat. The retention period will be until the later date of either 5 years after termination or completion of the entire trial or 3 years after the final report of the trial results.

### 9.4.4 Imaging CRO

Documents or records with regard to this trial will be properly stored in accordance with the SOPs of the imaging CRO. The retention period will be until the later date of either 5 years after termination or completion of the entire trial or 3 years after the final report of the trial results.

## 9.5 Record Retention and Destruction of Provision of Information

Although information will not be provided to other research institutes outside of this research organization in this trial, the CRO will collect information as an outsourced service related to the trial. Therefore, from the viewpoint of traceability, the CRO will keep the following records concerning the provision of information. These records will be disposed as described in Section 9.4.3.

| | |
|---|---|
| Name of an institute to which information is provided | See Section 15 “Trial Structure, Supportive Secretariat” |
| Name of an investigator in the institute to which information is provided | See Section 15 “Trial Structure, Supportive Secretariat |
| Name of institutes providing the information | See “Annex 1, List of Trial sites and Investigators” |
| Name of an investigator in the institutes providing the information | See “Annex 1, List of Trial sites and Investigators” |
| Information Items | See Section 9.2.1, ”Input Items of eCRF” |
| Course of Information collection | Collected at each trial site in accordance with the trial protocol. |
| Names of trial subjects | Retention of names of trial subjects will be substituted by retaining the consent forms appropriately at each trial site. |

# 10. Ethical Considerations

## 10.1 Protection of Trial Participants

This trial will be conducted in compliance with the "Declaration of Helsinki" (translated by the Japan Medical Association)*, the "Clinical Trials Act" (Act No. 16, 2017)**, "Ordinance for Enforcement of Clinical Trials Act" (Ministerial Ordinance No. 17, Ministry of Health, Labor and Welfare of 2008)**, "Ethical Guidelines for Medical and Health Research Involving Human Subjects" (Notification No. 1, Ministry of Education, Culture, Sports, Science and Technology, Ministry of Health, Labor and Welfare, Ministry of Economy, Trade and Industry, March 23, 2021, partial revision on March 10, 2022)***, related notices, and this trial protocol.

- * http://dl.med.or.jp/dl-med/wma/helsinki2013j.pdf
- ** http://www.mhlw.go.jp/stf/seisakunitsuite/bunya/0000163417.html
- *** https://www. mhlw.go.jp/content/000909926.pdf

## 10.2 Informed Consent

### 10.2.1 Provision of Information and Consent

The investigator or co-investigator will explain the following items using the informed consent document before a subject participates in the trial. The investigator or co-investigator should give a potential subject opportunities for questionning and counselling, and sufficient time and ask him/her to participate in the trial after confirming that he/she fully understands the content of the trial. If a potential subject agrees to participate in the trial, obtain the subject's signature on the consent form. Regardless of whether the subject is enrolled in the trial or not, the consent document of the subject will be kept at the trial site, and a copy of the consent document will be given to the subject. In a case that a patient is unable to read a document due to visual impairment, etc. but is able to understand its contents through oral explanation, or a case that a patient is unable to sign a document due to limb impairment, etc. but is able to read and understand the contents of the document), a witness must be present for the explanation and consent. The witness must sign and date the consent document and certify that the prospective subject understands this study and has given his/her consent freely and voluntarily. The witness must not be a person engaged in this research.

1) The name of the trial and the fact that the trial is conducted with permission of the administrator of the trial site and that the Trial Plan has been submitted to the Minister of Health, Labour and Welfare
2) A name of the trial site and the investigator, title, and contact address
   When a specified clinical trial is conducted as a multicenter trial, the name and title of the principal investigator, the names of other trial sites, and the names and titles of the investigator of the trial sites should be included.
3) Reasons why he/she was selected as a subject of the specified clinical trial
4) Benefits and risks expected by conducting the specified clinical trial
5) It is voluntary to refuse participation in the specified clinical trial.
6) Matters concerning withdrawal of consent
7) Refusal to participate in the specified clinical trial or withdrawal of consent will not result in mistreatment.
8) Methods for disclosing information on the specified clinical trial

9) A subject can obtain or view the trial protocol and other materials related to conduct of the specified clinical trial at the request of the subject. Methods of obtaining or viewing such materials.
10) Matters concerning the protection of the personal information of the subjects of the specified clinical trial
11) Methods of storage and disposal of trial materials
12) Sources of funding for the trial, conflicts of interest related to the trial in a trial site and personal earnings, and the status of conflicts of interest related to the trial of the investigators, etc.
13) A system related to responses to complaints and inquiries
14) Matters concerning costs related to conduct of the specified clinical trial
15) Presence or absence and details of other treatments and comparison of benefits and risks with those expected for other treatments
16) Matters concerning compensation and provision of medical care for health damage caused by conduct of the specified clinical trial
17) Matters to be reviewed by the CRB which is responsible for reviewing the specified clinical trial, and other matters related to the CRB for this specified clinical trial.
18) Other matters necessary for conduct of the specified clinical trial (purpose, significance, methods, duration of the trial, secondary use of data, etc.)

#### 10.2.2 Withdrawal of Consent

Withdrawal of consent means withdrawal of consent to participate in the trial, and is to be distinguished from refusal to continue treatment (① below). When a subject expresses withdrawal of consent, the investigator or co-investigator should clarify if the case is either (2) or (3) below and entered in the "Discontinuation/Completion Report" page in the eCRF.
In case of withdrawal of consent (② below), the investigator or co-investigator will not request for follow-up according to the trial protocol. In the case of withdrawal of entire consent (③ below), once it is confirmed that a patient requests withdrawal of entire consent, the data of the patient will not be viewable, modified, output, or analyzed.
Procedures for discontinuation of the request for follow-up of the patient and removal of patient data from the analysis will be defined separately.

① Refusal to continue the treatment: refusal to continue the further treatment (follow-up will continue).

② Withdrawal of consent: withdrawal of consent to participate in the trial, and refusal of all subsequent treatment and follow-up in accordance with the trial protocol. The data collected prior to the withdrawal of consent can be used.

③ Withdrawal of entire consent: withdrawal of consent to participate in the trial and disallowing research use of all data from the time of participation in the trial, including information at the time of enrollment. However, if the data has already been locked and reported in an analytical report, it cannot be withdrawn.

### 10.3 Patient consultation

The investigator or co-investigator will set up a consultation service for this trial from the trial subjects and the person concerned, and will describe the contact method in the informed consent

document. If a patient or his/her family member asks for consultation regarding this trial after enrollment, in principle, the researcher (the investigator or co-investigator) at the trial site where the patient visit will provide consultation to him/her. If the method of response is unclear, the investigator or co-investigator will discuss it with the Principal Investigator and Research Executive Committee members through the Supportive Secretariat as necessary, and decide on an appropriate response.

### 10.4 Protection of personal information and Identification of Patients

Names of the subjects will not be made known by the trial sites to any person in charge of the Supportive Secretariat, except for the person in charge of trial monitoring.
Subject identification and inquiries are made using the enrollment number and subject identification code issued at the time of enrollment. Information that would allow a third party to directly identify the subject (such as subject's name) without unauthorized access to the trial site's staff or database will not be entered into a database of this trial. When the results of this trial are made public, information that allows to identify a subject will never be disclosed.
For the use of personal information at the trial sites, the trial sites will give full consideration to protection of confidentiality of subject information to minimize the risk of information leakage, and the information will be managed appropriately in accordance with provisions at each trial site.

#### 10.4.1 Purpose of Use of Personal Information, Information to be Used, and Usage

(1) Purpose of Use

In accordance with the "To provide the best possible treatment to as many patients as possible," personal information of subjects will be used for the purpose of "To investigate specific subjects not only during treatment but also for a long period of time after the completion of treatment in order to obtain correct results of the clinical trial, and to properly manage the information obtained”.
Information from this research will be provided to F. Hoffmann-La Roche Ltd. (Switzerland). When the information is provided, consent will be obtained from the patients after explanation that appropriate measures in accordance with the laws and regulations* of the country to which the information will be taken.
* (Reference Information) Home page of the Personal Information Protection Commission, Regulations outside Japan
https://www.ppc.go.jp/enforcement/infoprovision/laws/

(2) Information to be Used

The following items considered minimum necessary for subject identification or inquiries will be used.

・Subject identification number (assigned at trial sites), date of birth

Thus, personal information other than the above, such as the names of trial subjects, will not be informed to the data center by the trial sites.

(3) Usage

The investigator, co-investigator, or clinical research coordinator at the trial sites will collect subject's personal information and medical information and enter such

information in the eCRF. Personal information will not be exchanged via e-mail. Although there may be cases in which data such as medical records are directly viewed through central monitoring based on data entered in the eCRF and on-site visits, even if personal information is accessed for the purpose of such monitoring and audits, there is no risk of such information being disclosed to outside parties due to contractual agreements and confidentiality obligations.

#### 10.4.2 Secondary Use of Data

Data and samples collected in this trial may be secondarily used in Japan or overseas if such secondary use is reviewed by the Principal Investigator and Collaborative Institute, and approved by an institutional review board. In such cases, appropriate measures will be taken in accordance with relevant domestic and foreign regulations. When the principal investigator or the collaborative institute provides the clinical research data to third parties (other medical institutes, pharmaceutical companies, etc.), including foreign countries, the data should be handled so that individuals cannot be identified.

#### 10.4.3 Response to a Request for Information Disclosure

When a subject of this trial requests the disclosure of personal information that identifies him/her, the relevant personal information retained at the trial site will be disclosed in principle. However, if disclosure may harm the life, body, property, or other rights or interests of the subject or a third party, or may seriously impede the proper execution of the work of the researchers involved in this trial, all or part of the personal information may not be disclosed. When a decision is made not to disclose all or part of the personal information requested for disclosure, the subject will be notified to that effect and the reasons must be explained.

#### 10.4.4 Information Management System

Various safety control measures will be taken to minimize the risk of information leakage when using personal information.

### 10.5 Need and System for Genetic Counseling

Since this trial will not deal with genetic information, genetic counseling will not be provided.

### 10.6 Compliance with Trial Protocol

Researchers participating in this trial will comply with this trial protocol as long as it does not compromise the safety and human rights of subjects.

### 10.7 Approval by CRB and Notification to MHLW

Before conducting this trial, the Principal Investigator must obtain approval from the CRB and permission to conduct the trial from the administrators of each trial site, using the trial protocol and the informed consent document. In addition, prior to the commencement of the trial, the Principal Investigator must submit the Trial Plan* to the Minister of Health, Labour and Welfare and publish the trial information in the jRCT. The Principal Investigator is responsible for the application to the CRB, submission of the Trial Plan to the Minister of Health, Labour and Welfare, and registration in the jRCT, and the Supportive Secretariat will assist the Principal Investigator in these application procedures.

* Form No. 1, specified in Article 39, Paragraph 1 of the Ordinance for Enforcement of the Clinical Trials Act

### 10.7.1 Procedure for New Application

<Procedures from an initial application to trial initiation>

1. The Principal Investigator will prepare the documents required by the CRB for this trial and submit them to the CRB for review.
2. The Principal Investigator will revise the trial protocol and informed consent documents as necessary in response to the review comments provided from the CRB.
3. The Principal Investigator will obtain permission to conduct the trial from the administrator of a trial site to which he/she belongs using the CRB review result notification and the documents submitted in 1), and send them to the investigators of the trial sites listed in the Trial Plan, and request approval from the administrators of each trial site.
4. The Supportive Secretariat will make a request for registration in the jRCT once it has obtained permission from the administrators of the trial sites described in the Trial Plan.
5. After registration in jRCT is completed, the Principal Investigator will print out the Trial Plan from jRCT, affix a seal, and submit it to the Minister of Health, Labour and Welfare (the regional health bureau with jurisdiction over the location of the CRB. The same shall apply hereinafter).
6. When the submission is accepted and the status of the jRCT is updated from "Registration Pending" to "Registration Open," the Principal Investigator will notify the CRB of it. The Supportive Secretariat will open the enrollment system and announce the start of the trial to the trial sites. jRCT's registration open date will be the start date of this trial.

Changes to "Section 15 Trial Structure" (addition or replacement of a trial site) fall under the category of changes to the content of the trial protocol, so the change procedure will be performed in accordance with "Section 10.8 Procedures for Application of Amendment".

### 10.7.2 Procedure to be Performed by Investigators at Each Trial Site

After the approval by the CRB is obtained, the investigators at each site will obtain approval from the administrator of a trial site to which he/she belongs. After obtaining the administrator's approval, the investigator will report it to the Supportive Secretariat as soon as possible.

### 10.7.3 Approval of Trial Conduct at Each Trial Site

Procedures to obtain permission to conduct the trial from the administrator of the trial site to which the investigator belongs is in accordance with the regulations of each trial site.
Note that changes to the trial protocol and the informed consent document approved by the CRB are not permitted, except for the contact information of the trial site and the pre-specified optional items. If changes to the content of the trial protocol or the informed consent documents are necessary and the administrator of the trial site requests modification, the investigator should consult with the Principal Investigator and the Supportive Secretariat.

## 10.8 Procedure for Application of Amendment

### 10.8.1 Procedure to be Performed by Principal Investigator

If it is necessary to change the content of materials submitted to the CRB for a new application, the Principal Investigator will submit a review request regarding the change to the CRB.
If the contents of changes involve a change in the Trial Plan, such changes should be registered in the jRCT and notified to the Minister of Health, Labour and Welfare. For a matter with regard to the progress, it should be done without delay after the change, and for other matters, it should be done after the Principal Investigator obtains approval from the CRB before implementation of the change. The Principal Investigator will promptly notify the CRB, administrators of the trial sites to which the investigators belong, and the investigators of that effect.
Procedures for the change application to the CRB follow 2), 3), and 4) of "Procedures from an initial application to trial initiation" in the previous section. In the case of changes that do not result in a change in the Trial Plan, the Supportive Secretariat will make a request for a report to the administrators of each trial site in the Step 4).
For minor changes defined in the ministerial ordinance, the Principal Investigator will notify the CRB of the details of the change within 10 days of the date of the change, register the change in the jRCT, and report the change to the Minister of Health, Labour and Welfare. If the trial is no longer continued at some trial sites, the changes to the Trial Plan will be submitted after the observation period for subjects is completed at the sites.
In the case of modifying the trial protocol, the Management Standards of Conflict of Interest or the Management Plan of Conflict of Interest, the opinions of the certified review board must be obtained in advance because of the possibility of modification of the implementation plan. As a result, if the implementation plan is to be modified, the modified implementation plan and a notification form according to Form 2 must be submitted to the MHLW.
When the planned statistical analysis is changed, the trial plan (or the statistical analysis plan if it has been prepared) should be revised, and it should be explained also in the clinical study eport.

### 10.8.2 Procedure to be Performed by Investigators at Each Trial Site

When the investigator of each trial site receives information on approval from the CRB from the Principal Investigator, he/she will obtain approval from the administrator of a trial site to which he/she belongs. The investigator of each trial site will promptly report to the Supportive Secretariat after obtaining the approval of the administrator.
For minor changes not including a change in the Trial Plan, the investigator of each trial site will report it to the administrator of the trial site.
If there is any change in the organization of the trial site to which the investigator belongs, the investigator of each trial site should inform the Principal Investigator and the Supportive secretariat, preparing documents concerning conflicts of interest and a list of co-investigators as necessary.

### 10.8.3 Review and Approval of Progress of Trial or Trial Continuation (Periodical Report)

The Principal Investigator will report to the administrators of the trial sites on the implementation status of 1) to 5) below, and submit the periodic reports to the CRB for review as to whether or not continuation of the trial is appropriate. The Principal Investigator will report to the Minister of Health, Labour and Welfare within one month from the date when the CRB provides opinions and within two months after the end of the relevant period every year starting from the date of

submission of the first Trial Plan. When a report is made to the CRB, the Principal Investigator will promptly provide information to the investigators of each trial site, and the investigators of each trial site will report to the administrators of their respective sites.

1) Number of subjects in this trial (planned number of patients, number of patients who provided consent, number of patients enrolled, number of patients who completed the trial, number of patients who discontinued the trial, and number of cases compensated)
2) Status of occurrence of “disease or like” related to this trial and its subsequent course (for details, refer to the "Procedures for Handling the Occurrence of Disease or Like" to be prepared separately)
3) Occurrence of non-compliance with the ministerial ordinance pertaining to this clinical trial or the trial protocol and subsequent responses
4) Evaluation of the safety and scientific rationale of the trial
5) Any applicable matter if there was a change in the Management Standards of Conflict of Interest or the Management Plan of Conflict of Interest for this trial.

#### 10.8.4 Reporting of Progress of Clinical Trial

The Supportive Secretariat will maintain information on the progress of the trial and the occurrence of “disease or like” and defects, as appropriate. The Supportive Secretariat will provide the Principal Investigator with the necessary information for the processes of the periodic reporting.

### 10.9 Conflict of Interest

#### 10.9.1 Managing Conflict of Interest Related to This Clinical Trial

Conflicts of interest related to this trial will be managed as follows in accordance with the "Guidance for the Management of Conflicts of Interest in Clinical Trials Act" (Notification of the Director, Research and Development Division, Health Policy Bureau, Ministry of Health, Labour and Welfare, November 30, 2018, No. 1130-17) * (hereinafter referred to as the Guidance).

※http://www.mhlw.go.jp/stf/seisakunitsuite/bunya/0000163417.html

The forms to manage conflicts of interest in the latest version of the guidance should be used.

- Management Standards for Conflict of Interest: Form A
- Relevant Company Report: Form B
- Researcher’s self-declaration form Conflict of Interest: Form C
- Status Confirmation Report for Conflict of Interest: Form D
- Management Plan of Conflict of Interest: Form E

Among the investigators and co-investigators of this trial, for researchers who meet the Criterion 1: ②A, C[#1] or Criterion 4: ②[#2] of the Management Standards for Conflict of Interest (Form A), it will be disclosed that they meet the criteria at the time of publication of the trial results. In addition, the investigators who fall under "Criterion 4:②[#2]" shall undergo an audit during the trial period. Even in this case, the investigators shall not engage in any work involving data management, monitoring, and statistical analysis.

| | | |
|---|---|---|
| #1 | Criterion 1 : ②A | Donations of more than total 2,000,000 yen per year (the total amount received, including indirect expenses, as long as the use of the donation is practically determinable. The same shall apply hereinafter.) from the relevant pharmaceutical company to a person who will clearly benefit by conducting the clinical trial (hereinafter referred to as "Conflict of Interest Declarant") among the investigator, co-investigator, responsible statistician, and other persons listed in the trial protocol. |
| | Criterion 1 : ②C | The Conflict of Interest Declarant or his/her spouse or first-degree relative (parent or child) who shares the same livelihood with the Conflict of Interest Declarant (hereinafter referred to as "Conflict of Interest Declarant, etc.") who receives total annual personal profits (income from salary, lectures, manuscript writing, consulting, intellectual property rights, gifts, hospitality, etc. The same shall apply hereinafter) of 1,000,000 yen or more from the relevant pharmaceutical company. |
| #2 | Criterion 4 : ② | A researcher who receives a total annual personal profit of 2.5 million yen or more from the relevant pharmaceutical company. |

10.9.2 Funding Source of Clinical Trial/Funding, and Financial Relationships

This trial will be funded by Chugai Pharmaceutical Co., Ltd. based on an agreement between Shinshu University Hospital, to which the Principal Investigator belongs, IQVIA Services Japan G.K. to which some of the trial operations are entrusted, and Chugai Pharmaceutical Co. Ltd. The trial sites will enter into a subcontracting agreement with IQVIA Services Japan G.K. and will receive research funding based on the number of enrollments and other clinical trial tasks. The funds are not intended to promote patient enrollment, but rather to compensate for the workload involved in the clinical trial. The trial will be conducted using pharmaceutical products manufactured and marketed by Chugai Pharmaceutical Co., Ltd., however, Chugai Pharmaceutical Co., Ltd. will not be directly involved in the monitoring, data management, statistical analysis, or auditing of this trial, and is not in a position to influence the trial results.

10.9.3 Information Disclosure

Information on the provision of research funds related to this trial is subject to public disclosure. Provision of such information to universities and other institutions to which the relevant persons belong is also subject to disclosure. Such information will be disclosed on the website of Chugai Pharmaceutical Co. Ltd. after the end of each fiscal year.

10.10 Trial Expense

10.10.1 Expense for Treatment

This clinical trial will be covered by the usual health insurance. Observations and tests during the trial period and drugs used will be covered by the subject's health insurance, and will be paid for by the subject within his/her insurance coverage (Transportation expenses to visit a site will also be paid by patients). However, participation in this study may result in an increase in the number of visits and examinations compared to normal clinical practice, and an increase in the burden of examination costs. Therefore, patients will be charged 3,000 JPY per visit (up to 7,000 JPN if

transportation expenses exceed 3,000 JPN) for each visit at which observation and examination are scheduled without research drug administration, and 1,000 JPN for each response to the questionnaire as a reimbursement. In addition, 10,000 JPN will be paid to patients who exceed a certain level of input for daily subjective symptom surveys using the application.

#### 10.10.2 Compensation for Health Damage

When an adverse event occurs during the trial, the investigator will promptly take the necessary action (examination, treatment, or discontinuation of the trial, etc.) to ensure the safety of the subject. In such cases, the best possible medical care within the scope of insurance treatment shall be provided. The Principal Investigator and the Collaborative Institute shall take necessary measures such as obtaining clinical trial compensation insurance prior to the start of the trial to prepare for legal liability and compensation for health damage resulting from the clinical trial.
In addition, the investigator will ensure that a system is in place to provide medical care and take other necessary measures against health hazards arising from the conduct of this trial.

## 11. Record、Monitoring, and Audit of Trial

### 11.1 Record of Trial

The investigators must keep sufficient and accurate records, including the trial protocol, informed consent documents, and approval documents from the administrators of the study sites , in order to completely document the implementation status of the trial.

### 11.2 Monitoring

Designated persons of the Support Secretariat will monitor the trial in order to confirm that the trial is being conducted safely and in accordance with the trial protocol, and that data are being collected accurately. The monitoring will be performed by central monitoring based on the data entered in the eCRF accumulated at the data center. In addition, the monitoring shall be conducted in accordance with the Monitoring Plan to be prepared separately.

#### 11.2.1 Central Monitoring

Central monitoring will be performed based on the data entered in the eCRF to ensure that the trial is being conducted safely and in accordance with the trial protocol, and that data are being collected accurately. In principle, central monitoring will be performed twice a year (considering from the first patient enrollment), and the monitors of the Supportive Secretariat will prepare periodical monitoring reports and submit them to the Principal Investigator and the Responsible Research Institute. Periodical monitoring reports will be reviewed by the Principal Investigator and may be used for feedback and discussion of problems. Monitors of the Supportive Secretariat will provide information on the contents of the periodical monitoring reports to the investigators, according to instructions of the Principal Investigator. The investigators at the trial sites may use periodical monitoring reports for periodical reporting to the administrators of trial sites. The items of the periodical monitoring report are as follows.

1) Enrollment status: Number of enrolled patients—cumulative/by period/by site
2) Eligibility of subjects (ineligible/potentially ineligible subjects): Trial site
3) Treatment discontinuation: Reason/Situation
4) Deviation from the trial protocol: Trial site
5) Serious adverse events: Trial site

6) Adverse reactions/Adverse events
7) Other issues relating to the progress of the trial or safety

11.2.2 On-site Monitoring
The frequency and details of the procedures for on-site monitoring will be in accordance with the Monitoring Plan to be prepared separately. The Collaborative Institute will not conduct verification of source documents containing subjects' personal information by on-site visits.

11.2.3 Audit
Based on the results of the central monitoring, the Supportive Secretariat will consider conducting on-site audits to improve the scientific and ethical quality of the trial. Procedures, including the items to be checked and the frequency of the audits in the on-site audits will be specified separately. The auditors of the Supportive Secretariat engaged in the audit will report the audit results to the investigator. The auditors will provide the information to other Investigators as necessary according to the direction of the Principal Investigator.

11.3 Violation and Deviation from Trial Protocol
Violations are any actions that were not performed in accordance with the provisions of the Ordinance for Enforcement of the Clinical Trials Act, the trial protocol etc. Deviations from the trial protocol within acceptable limits established in advance or post hoc by the principal investigator will not be considered violations.

1) Violation

Failure to comply with the Ordinance for Enforcement Regulations of the Clinical Trial Act, trial protocols, procedure manuals, etc., falsification or fabrication of research data.

2) Important violation

Violation that which affects the human rights or safety of the subjects of a clinical trial, progress of the trial, or the reliability of the results. For example, it refers to non-compliance with inclusion/exclusion criteria, discontinuation criteria, concomitant use of prohibited therapies, etc. Failures to comply with the trial protocol to avoid immediate hazard to the subjects or for any other compelling medical reasons are excluded.

When the investigator or co-investigator notices that this trial is non-compliant, he/she will promptly inform an administrator of the trial site (in the case of a co-investigator the investigator or an administrator of the trial site), and the principal investigator. When the principal investigator notices that the trial is noncompliant, he/she will promptly provide information to the other investigators to that effect. If any of the nonconformities are found to be an important violation, the principal investigator will promptly obtain the opinion of the CRB.

3) Acceptable Deviation

Deviations from the trial protocol that are within acceptable limits specified by the Principal Investigator in advance or post hoc.

## 12. Publication of Data

## 12.1 Record to the Public Database

Disclosure of Implementation Plan

Information on this trial will be made public by recording it in advance in the database (jRCT: Japan Registry of Clinical Trials) maintained by the Ministry of Health, Labour and Welfare (MHLW). Any changes to the implementation plan will also be made public by recording the revised information in the same manner.

1. Publication of the primary endpoint report

When the data collection period for the primary endpoints described in the trial protocol has been ended at all domestic and overseas sites, the principal investigator will prepare a primary endpoint report (a summary of the results of data collection on the primary endpoints) in principle within one year from that date, and also make changes to the implementation plan. The investigator will obtain the opinions of the certified review board on the primary endpoint report and the modified implementation plan in advance, submit them to the administrator of the trial site without delay, and make them public by recording them in the jRCT within one month from the date when the certified review committee gives its opinions.

2. Publication of a summary of a clinical trial report

When the data collection period for all evaluation items described in the trial protocol has been ended at all domestic and overseas sites, the principal investigator will prepare a clinical trial report (a document summarizing the results of this trial) and its summary (a summary of the trial results in the jRCT is also acceptable) in principle within one year from that date. The summary report should include at least the following (1) to (2). The summary report should include at least the following items (1) to (4).

(1) Background information on the subjects of the clinical retrial (age, gender, etc.)
(2) Information on the progress depending on the design of the clinical trial (e.g., progress in the number of subjects enrolled)
(3) Summary of the occurrence of diseases, etc.
(4) Data analysis and results of the primary and secondary endpoints

The principal investigator will obtain the opinions of the certified review board on the prepared clinical trial report and its summary in advance, and submit it to the administrator of the trial site without delay. The summary of the clinical trial report will be made public by recording it in the jRCT within one month of the date on which the certified review board expressed its opinion. In addition, the trial protocol, informed consent documents, and statistical analysis plan (if prepared) must be submitted to the MHLW along with the summary of the clinical trial report. In cases where the primary endpoint report and the clinical trial report must be prepared at the same time, the primary endpoint report can be deemed to have been prepared by the preparation of the clinical trial report.

## 12.2 Publication of Data

When the results of this trial are to be presented in a paper or at an academic conference, the Principal Investigator, Investigator, and Collaborative Institute will discuss to reach an agreement.

### 12.3 Layperson Summary (LPS)

After the publication of a main paper, a Layperson Summary (LPS) will be prepared based on the trial results and data published in the paper to explain the trial results to the subjects who participated in the trial and their families. The LPS will be used by the researcher to explain the trial results to the subject only when the researcher is asked by the subject to explain the trial results to the subject.

## 13. Discontinuation, Interruption, or Termination of Entire Trial

### 13.1 End of Entire Trial

After the Responsible Research Institute determines completion or discontinuation of the entire trial and a clinical trial report is approved by the Responsible Research Institute, the Responsible Research Institute will notify the Principal Investigator of the end of the entire trial.

### 13.2 Discontinuation or Interruption of Entire Trial

If it is necessary to take urgent safety measures such as withdrawal of the marketing authorization of the IMP or distribution of urgent safety information (yellow letter) due to unexpected serious adverse events, the Responsible Research Institute will make a decision to discontinue or suspend the entire trial after confirming the medical and ethical opinions of the Research Executive Committee members. If it is determined during the course of the trial that the entire trial must be discontinued or suspended, the Responsible Research Institute will promptly notify the administrators of the trial sites in writing of the discontinuation or suspension of the trial and details of the reasons for it. The administrators of the trial sites will notify the investigator and the CRB and explain the details in writing. The investigator or co-investigator will notify the subject and ensure appropriate treatment and post-treatment.

### 13.3 Clinical Trial Report

Upon receiving notification of the completion or discontinuation of the trial, the medical writing department of the Supportive Secretariat will prepare a clinical trial report summarizing the results of the trial and submit the draft report to the Responsible Research Institute without delay. The results will be interpreted and finalized by the Responsible Research Institute.

### 13.4 Procedure at the End of Trial

After confirming the completion of the trial, the Principal Investigator will report to the investigators of the trial sites that the trial has been completed, together with a summary of the results. The timing of distribution of the summary of trial results (the trial completion report) will be decided by the Responsible Research Institute and the Principal Investigator, taking into consideration the timing of publication of the main results. The trial completion report will be distributed to the investigators of the trial sites through the Supportive Secretariat. Upon receiving the trial completion report, the investigators of the trial sites will promptly go through the procedures for completion in accordance with the rule of each trial site. For a trial site without patient enrollment, the date of completion of the trial may be the date of the end of enrollment at the trial site.

## 14. Attribution of Trial Results

Trial results generated from this clinical trial shall belong to the Collaborative Institute, Chugai Pharmaceutical Co. Ltd. Inventions as trial outcomes using the obtained data, as well as patents, improvements, know-how, etc. derived therefrom, shall be managed as the exclusive and free property of Chugai Pharmaceutical Co. Ltd. Intellectual property rights for the pharmaceutical products manufactured and sold by Chugai Pharmaceutical Co. Ltd.

## 15. Trial Structure

### 15.1 Principal Investigator

Department of Ophthalmology, Shinshu University School of Medicine, Professor, Toshinori Murata
3-1-1 Asahi, Matsumoto Nagano 390-8621
Tel. 0263-37-2789

<Role of Principal Investigator>
Responsible for coordinating the entire trial organization including all trial sites involved in this trial, and for ensuring the trial being conducted in compliance with the Clinical Trials Act.

### 15.2 Responsible Institute/Collaborative Institute

Chugai Pharmaceutical Co., Ltd.
2-1-2 Nihonbashi-Muromachi, Chuo-ku, Tokyo 103-8324
Tel. 03-3273-0866

#### 15.2.1 Representative Director of Responsible Institute/Collaborative Institute

Chugai Pharmaceutical Co., Ltd. Representative Director, Osamu Okuda

<Role and Responsibility of Representative Director of Responsible Institute/Collaborative Institute>
To supervise that this trial is properly conducted at the Collaborative Institute in accordance with the "Clinical Trials Act", the “Ethical Guidelines for Medical and Health Research Involving Human Subjects", and the trial protocol. To take appropriate measures in case of any violation of the internal rules of the Collaborative Institute and the trial protocol.

#### 15.2.2 Division Director of Responsible Institute/Collaborative Institute

Chugai Pharmaceutical Co., Ltd. Medical Affairs Division General Manager, Kaori Ouchi
2-1-1 Nihonbashi-Muromachi, Chuo-ku, Tokyo 103-8324
Tel. 03-3273-0866

<Role and Responsibility of Division Director of Responsible Institute/Collaborative Institute>

(1) To enter into an agreement for funding for this trial.
(2) Overall supervision of the trial
    1) Shall be responsible for providing the necessary supervision to ensure that the trial is conducted properly at the Collaborative Institute.

2) To confirm as necessary that the trial is being conducted properly at the Collaborative Institute in accordance with the aforementioned guidelines and trial protocol, and take necessary measures to ensure the proper conduct of the trial.
3) To ensure that all persons involved in the implementation of the trial who are authorized to be conducted at the Collaborative Institute are informed that the trial must be conducted with respect for the life, health, and human rights of the trial subjects and others involved, and that disciplinary or other adverse actions may be taken if they violate the internal rules of the Collaborative Institute or trial protocol.
4) Not to leak information obtained in the course of his work without legitimate reasons. The same shall apply even after he/she ceases to be engaged in the work.
5) To conclude a written agreement on matters to be observed by the entrusted party when outsourcing a part of the work in the trial authorized to be conducted at the Collaborative Institute, and to exercise necessary and appropriate supervision over the entrusted party.

(3) Development of a system and regulations to perform operations of this trial.

1) To ensure that appropriate measures to compensate and other necessary measures are taken if a subject suffers a health hazard relating to the trial authorized to be conducted at the Collaborative Institute.
2) To ensure that the results of the trial and other information about the trial are appropriately made public, after taking the necessary measures to protect the human rights of the subjects and other persons concerned, and the rights and interests of the researchers and other persons concerned.
3) To conduct self-checks and evaluations as necessary if the trial authorized to be conducted at the Collaborative Institute conforms to the internal rules of the Collaborative Institute, and to take appropriate actions based on the results of such checks and evaluations.
4) To take measures to ensure that the researchers and other persons concerned involved in the trial authorized to be conducted at the Collaborative Institute receive education and training with regard to knowledge and techniques necessary for conducting the trial. The Division Director must also receive such education and training.

15.2.3 Medical Expert and Responsible Person in Responsible Institute/Collaborative Institute

Medical expert in the collaborative Institute

Chugai Pharmaceutical Co., Ltd. Specialty Medical Science Department, Associate Medical Manager, Takashi Omoto

Responsible person in the collaborative Institute

Chugai Pharmaceutical Co., Ltd. Specialty Medical Science Department, Medical Manager, Jun Tsujimura
1-1 Nihonbashi-Muromachi 2-Chome, Tokyo 103-8324
Tel. 03-3273-0866

<Role and Responsibility of Medical Expert and Responsible Person in Responsible Institute/Collaborative Institute>

- To collaborate with the Principal Investigator to develop the Trial Plan.
- A responsible person in the Collaborative Institute is responsible for properly conducting the tasks assigned to the Collaborative Institute in this trial in accordance with the internal regulations and trial protocol.
- Responsible for execution of the duties as a medical expert in this trial and for ensuring the proper conduct of this trial based on medical point of views.

## 15.3 Sponsor

Chugai Pharmaceutical Co., Ltd. Medical Affairs Division General Manager, Kazuhiko Nishi
2-1-1 Nihonbashi-Muromachi, Chuo-ku, Tokyo 115-8543
Tel. 03-3273-0866

<Role and Responsibility of Sponsor>
To fund this trial, properly disclose information, and bear the responsibility as a co-investigator of this trial.

## 15.4 Research Executive Committee (order of the Japanese syllabary)

Department of Ophthalmology, Kurume University School of Medicine
Professor, Shigeo Yoshida
Department of Ophthalmology, Juntendo University Graduate School of Medicine,
Professor, Shintaro Nakao

<Role of Research Executive Committee >

- Determination of medical judgment regarding the conduct of the trial
- Advice/recommendation on preparation/revision of a trial protocol
- Advice/recommendation on preparation/revision of an informed consent document (sample)
- Advice/recommendation on the design and preparation of an eCRF
- Determination of candidate trial sites participating in this trial
- Checking the progress of the trial
- Preparation and dissemination of an eCRF input promotion letter
- Reviewing a publication plan
- Reviewing a statistical analysis plan
- Endorsement of a clinical trial report
- Sharing of information to the investigators of each trial site
- Other tasks as discussed with the Responsible Research Institute

## 15.5 Research Secretariat

Department of Ophthalmology, Shinshu University School of Medicine, Associate Professor, Takao Hirano
3-1-1 Asahi, Matsumoto Nagano 390-8621
Tel. 0263-37-2789

<Role of Research Secretariat>
To provide correspondence regarding the trial protocol, which involves medical judgment.

### 15.6 Trial sites

The Responsible Research Institute will select trial sites. The selected trial sites will consider whether or not to participate in the trial. Refer to Attachment 1 for the participating trial sites (Other sites are planned to participate later.).

### 15.7 Responsible Statistician

IQVIA Services Japan G.K.
Real World & Analytic Solutions (RWAS)
Real World Evidence Services, Data center, Biostatistics, Naoya Oishi
Keikyu Dai-1 Building 4-10-18 Takanawa, Minato-ku, Tokyo 108-0074
Tel. 03-6859-9500

<Role of a Responsible Statistician>
Management and supervision of statistical analysis work
Certification of validity of analysis results

### 15.8 Supportive Secretariat

IQVIA Services Japan G.K.
Keikyu Dai-1 Building 4-10-18 Takanawa, Minato-ku, Tokyo 108-0074
Tel. 03-6859-9500

<Role of Supportive Secretariat>
To provide support for trial operations as a contact for trial.

### 15.9 Organization Responsible for Monitoring

IQVIA Services Japan G.K.
Responsible Person: Gen Tomohiro
Keikyu Dai-1 Building 4-10-18 Takanawa, Minato-ku, Tokyo 108-0074
Tel. 03-6859-9500

<Role of an Organization Responsible for Monitoring>
To perform practical operations of trial monitoring

### 15.10 Organization Responsible for Data Management

IQVIA Services Japan G.K.
Responsible Person: Yusuke Nihei
Keikyu Dai-1 Building 4-10-18 Takanawa, Minato-ku, Tokyo 108-0074
Tel. 03-6859-9500

<Role of an Organization Responsible for Data Management>
To perform practical operations of data management, and to take responsibility for quality

assurance of data.

15.11 Organization Responsible for Audit
IQVIA Services Japan G.K.
Responsible Person: Katsura Nakamura
Keikyu Dai-1 Building 4-10-18 Takanawa, Minato-ku, Tokyo 108-0074
Tel. 03-6859-9500

<Role of an Organization Responsible for Audit>
To perform practical operations of a trial audit, and to take responsibility for audit results.

15.12 Person Supporting Research & Development Plan
IQVIA Services Japan G.K.
Responsible Person: Waka Shimomoto
Keikyu Dai-1 Building 4-10-18 Takanawa, Minato-ku, Tokyo 108-0074
Tel. 03-6859-9500

<Role of a Person Supporting Research & Development Plan>
To support the work to develop the most effective and efficient framework of a trial protocol based on a development plan of a pharmaceutical or other product.

15.13 Person in Charge of Coordination and Management
IQVIA Services Japan G.K.
Responsible Person: Waka Shimomoto
Keikyu Dai-1 Building 4-10-18 Takanawa, Minato-ku, Tokyo 108-0074
Tel. 03-6859-9500

<Role of a Person in Charge of Coordination and Management>
To manage the progress and budget of a clinical trial
To follow procedures necessary for clinical research, to appropriately manage documents, and to ensure the reliability of collected data
Liaison, coordination and exchange of information with parties involved in clinical trial

15.14 Imaging CRO
Micron Inc.
3-13-16 Mita, Minato-ku, Tokyo, 108-0073

<Role of Imaging CRO>
To perform practical operations of image analysis

15.15 ePRO Vendor
Integrity Healthcare Co., Ltd.
27-5, Nihonbashi Kakigara-cho 1-chome, Chuo-Ku, Tokyo 103-0014

<Role of ePRO Vendor>
To develop and provide the ePRO system used in this research

## 16. Reference


1. Miller K, Fortun JA. Diabetic macular edema: Current understanding, pharmacologic treatment options, and developing therapies. Asia Pac J Ophthalmol (Phila). 2018;7(1):28-35.
2. Romero-Aroca P. Managing diabetic macular edema: The leading cause of diabetes blindness. World J Diabetes. 2011;2(6):98-104.
3. 服部隆幸．糖尿病黄斑浮腫．湯澤美都子編．実践黄斑疾患．日本医事新報社．2016:238-52.
4. 安田美穂．久山町研究．あたらしい眼科　2016;33(9):5-10.
5. 難波広幸、川崎良、山下英俊．舟形町研究．あたらしい眼科　2016;33(9):11-18.
6. 川崎良、山下英俊．疫学に基づいた糖尿病網膜症の管理．　月刊糖尿病 2013; 5(1):23-29.
7. 糖尿病網膜症診療ガイドライン（第 1 班）．日本眼科学會雜誌 2020;124(12):947-949
8. バビースモ添付文書
   https://www.pmda.go.jp/PmdaSearch/iyakuDetail/ResultDataSetPDF/450045_1319408A1020_1_02
   最終アクセス日：2022 年 9 月 20 日
9. Lai TT, Chen TC, Yang CH, et al. Treat-and-extend vs. *pro re nata* regimen of ranibizumab for diabetic macular edema-A two-year matched comparative study. Front Med (Lausanne). 2022;8:781421.
10. Sarohia GS, Nanji K, Khan M, et al. Treat-and-extend versus alternate dosing strategies with anti-vascular endothelial growth factor agents to treat center involving diabetic macular edema: A systematic review and meta-analysis of 2,346 eyes. Surv Ophthalmol. 2022;67(5):1346-1363.
11. Volkmann, I., Knoll, K., Wiezorrek, M. et al. Individualized treat-and-extend regime for optimization of real-world vision outcome and improved patients' persistence. BMC Ophthalmol 2020;20:122.
12. Shimura M, Kitano S, Muramatsu D, et al. Real-world management of treatment-naïve diabetic macular oedema in Japan: two-year visual outcomes with and without anti-VEGF therapy in the STREAT-DME study. Br J Ophthalmol. 2020;104(9):1209-1215.
13. Sugimoto M, Tsukitome H, Okamoto F, et al. Clinical preferences and trends of anti-vascular endothelial growth factor treatments for diabetic macular edema in Japan. J Diabetes Investig. 2019;10(2):475-483.
14. Ehlken C, Ziemssen F, Eter N, et al. Systematic review: non-adherence and non-persistence in intravitreal treatment. Graefes Arch Clin Exp Ophthalmol. 2020; 258(10):2077-2090.
15. Peto T, Akerele T, Sagkriotis A, et al. Treatment patterns and persistence rates with anti-vascular endothelial growth factor treatment for diabetic macular oedema in the UK: A real-world study. Diabet Med. 2022; 39(4):e14746.
16. Wykoff CC, Abreu F, Adamis AP, et al. Efficacy, durability, and safety of intravitreal faricimab with extended dosing up to every 16 weeks in patients with diabetic macular

oedema (YOSEMITE and RHINE): two randomised, double-masked, phase 3 trials. Lancet. 2022;399(10326):741-755.

17. Grading diabetic retinopathy from stereoscopic color fundus photographs--an extension of the modified Airlie House classification. ETDRS report number 10. Early Treatment Diabetic Retinopathy Study Research Group. Ophthalmology. 1991;98(5 Suppl):786-806.
18. Mangione CM, Lee PP, Gutierrez PR, et al. Development of the 25-item National Eye Institute Visual Function Questionnaire. Arch Ophthalmol. 2001;119(7):1050-8.
19. Reilly MC, Zbrozek AS, Dukes EM. The validity and reproducibility of a work productivity and activity impairment instrument. Pharmacoeconomics. 1993;4(5):353-65.
20. バビースモ適正使用ガイド
    https://www.pmda.go.jp/RMP/www/450045/2e72f5fb-b81f-41ce-b2c7-d662f67b5d94/450045_1319408A1020_01_001RMPm.pdf
    最終アクセス日：2022 年 9 月 20 日
21. G regori NZ, Feuer W, Rosenfeld PJ. Novel method for analyzing snellen visual acuity measurements. Retina. 2010;30(7):1046-50.

Appendix 1 Schedule of Activities

**Extension-by-8W Regimen: Screening to W60**

| | | Screening | W0 | W4 | W8 | W12 | W16 | W20 | W24 | W28 | W32 | w36 | w40 | w44 | w48 | w52 | w56 | w60 |
|---|---|---|---|---|---|---|---|---|---|---|---|---|---|---|---|---|---|---|
| Visit Window [a] | | Day−28 ~ Day−1 | Day 1 | -7,+14 | -7,+14 | -7,+14 | -7,+14 | -7,+14 | -7,+14 | | -7,+14 | | -7,+14 | | -7,+14 | -7,+14 | -7,+14 | -7,+14 |
| Visit [b] | | x | x | x | x | x | x | x | x | | x | | x | | x | x | x | x |
| Informed Consent [c] | | x | | | | | | | | | | | | | | | | |
| Eligibility Check | | x | x | | | | | | | | | | | | | | | |
| Medical/Surgical History | | x | x | | | | | | | | | | | | | | | |
| Background Information | | x | | | | | | | | | | | | | | | | |
| Body Weight and Height | | x | | | | | | | | | | | | | | | | |
| Vital Signs [d] | | x | x | x | x | x | x | x | x | | x | | x | | x | x | x | x |
| Concomitant Medication and Therapy | | | x | x | x | x | x | x | x | | x | | x | | x | x | x | x |
| Adverse Event [e] | | | x | x | x | x | x | x | x | | x | | x | | x | x | x | x |
| Clinical Laboratory Test [f] | | x | | | | | | | | | | | | | | | x | |
| Intravitreal Administration of IMR [g] | | | x | x | x | | x | | | | x | | | | | | x | |
| Finger-counting Test [h] | | | x | x | x | | x | | | | x | | | | | | x | |
| Intraocular Pressure [i] | | x | x | x | x | x | x | x | x | | x | | x | | x | x | x | x |
| Refraction Test [j] | | x [j] | x [j] | | | | | | | | | | | | | | | |
| Visual Acuity Test [k,l] | | x | x | x | x | x | x | x | x | | x | | x | | x | x | x | x |
| Slitlamp Microscopy | | x | x | x | x | x | x | x | x | | x | | x | | x | x | x | x |
| Fundoscopy | Indirect Ophthalmoscopy | x | x | x | x | x | x | x | x | | x | | x | | x | x | x | x |
| | CFP [m] | ◎● | ◎● [n] | ● | ● | ◎● | ● | ● | ● | | ● | | ● | | ● | (●) [o] | (◎●) [o] | (●) [o] |
| | FA | x | x [n] | | | | | | x | | | | | | | (x) [o] | (x) [o] | (x) [o] |
| | ICGA | x [p] | x [p] | | | | | | x [p] | | | | | | | x [p] | x [p] | x [p] |
| Optical Coherence tomography | SD-OCT | x | x | x | x | x | x | x | x | | x | | x | | x | x | x | x |
| | OCT-A [l] | | x | | | x | | | | | | | | | | (x) [o] | (x) [o] | (x) [o] |
| ETDRS DRSS | | | x | | | x | | | | | | | | | | | x | |
| NEI-VFQ-25 [q] | | | x | | | x | | | | | | | | | | | x | |
| WPAI [q] | | | x | | | x | | | | | | | | | | | x | |
| Application for Patients [r] | | | ◄ | — | — | — | — | — | — | — | — | — | — | — | — | — | — | ► |

## Extension-by-8W Regimen: W64～W112

| | | W64 | W68 | W72 | W76 | W80 | W84 | W88 | W92 | w96 | w100 | w104 | w108 | w112 |
|---|---|---|---|---|---|---|---|---|---|---|---|---|---|---|
| Visit Window [a] | | -7,+14 | | -7,+14 | | -7,+14 | | -7,+14 | | -7,+14 | | -7,+14 | | -7,+14 |
| Visit [b] | | x | | x | | x | | x | | x | | x | | x |
| Informed Consent [c] | | | | | | | | | | | | | | |
| Eligibility Check | | | | | | | | | | | | | | |
| Medical/Surgical History | | | | | | | | | | | | | | |
| Background Information | | | | | | | | | | | | | | |
| Body Weight and Height | | | | | | | | | | | | | | |
| Vital Signs [d] | | x | | x | | x | | x | | x | | x | | x |
| Concomitant Medication and Therapy | | x | x | x | x | x | x | x | x | x | x | x | x | x |
| Adverse Event [e] | | x | x | x | x | x | x | x | x | x | x | x | x | x |
| Clinical Laboratory Test [f] | | | | | | | | | | | | | | x |
| Intravitreal Administration of IMR [g] | | | | | | x | | | | | | x | | |
| Finger-counting Test [h] | | | | | | x | | | | | | x | | |
| Intraocular Pressure [i] | | x | | x | | x | | x | | x | | x | | x |
| Refraction Test [j] | | | | | | | | | | | | | | |
| Visual Acuity Test [k,l] | | x | | x | | x | | x | | x | | x | | x |
| Slitlamp Microscopy | | x | | x | | x | | x | | x | | x | | x |
| Fundoscopy | Indirect Ophthalmoscopy | x | | x | | x | | x | | x | | x | | x |
| | CFP [m] | ● | | ● | | ● | | ● | | ● | | ● | | ◎● |
| | FA | | | | | | | | | | | | | x |
| | ICGA | | | | | | | | | | | | | x [p] |
| Optical Coherence tomography | SD-OCT | x | | x | | x | | x | | x | | x | | x |
| | OCT-A [l] | | | | | | | | | | | | | x |
| ETDRS DRSS | | | | | | | | | | | | | | x |
| NEI-VFQ-25 [q] | | | | | | | | | | | | | | x |
| WPAI [q] | | | | | | | | | | | | | | x |
| Application for Patients [r] | | ◄ | | | | | | | | | | | | ► |

CFP = color fundus photography, ETDRS DRSS = Early Treatment Diabetic Retinopathy Study Diabetic Retinopathy Severity Scale, FA = fluorescein angiography, HbA1c = hemoglobin A1c, ICGA = Indocyanine green angiography, NEI-VFQ-25 = The 25-item National Eye Institute Visual Function Questionnaire, OCT-A = Optical Coherence Tomography Angiography, SD-OCT = Spectral-Domain Optical Coherence Tomography, W = Week, WPAI = Work Productivity and Activity Impairment Questionnaire

Note: All ophthalmologic examinations should be performed on both eyes unless otherwise specified.

a. The date of the first dose is defined as Day 1, and the day before is defined as Day−1.

b. If active DME* is observed at W12 or later, the subsequent visit/dosing schedule should be changed (see Section 5.2). However, visits surrounded by bold lines should be executed regardless of a schedule change.

c. Consent from a patient must be obtained prior to conducting any trial-specific screening assessment. Consent may be obtained more than 28 days prior to the first dose of the IMP. For tests and evaluations performed as part of routine medical practices and conducted within 28 days of Day 1, available results can be used and there is no need to repeat such tests for screening even before consent is obtained.

d. Including systolic and diastolic blood pressure in the sitting position and pulse rate. An abnormal finding observed at baseline will be recorded in the eCRF as a complication. Any new clinically significant abnormal finding or clinically significant worsening of pre-existing abnormality observed at the subsequent visits will be recorded in the eCRF as an adverse event.

e. Patients will provide information on adverse events to investigators or co-investigators at each visit. Adverse events will be reported in the eCRF by investigators or co-investigators.

f. Measure HbA1c and serum creatinine

g. A dosing interval of the IMP should be ≥ 21 days.

h. A finger-counting test will be evaluated within 15 minutes after administration of the IMP to ensure that a patient has no visual function impairment.

i. Intraocular pressure of both eyes will be measured before eye dilation for ophthalmologic examination. If intraocular pressure is ≥ 30 mmHg, a mydriatic drug and IMP shouldn’t be administered. In addition, intraocular pressure of a study eye will be measured after 30 minutes post-dose whenever possible.

j. Spherical power, cylinder power, and cylinder axis will be recorded in the eCRF. The test on Day 1 is unnecessary if it was conducted at screening.

k. Performed prior to eye dilation.

l. A visual acuity test will be performed at a distance of 5 m using a Landolt ring chart.

m. Perform according to the manual for fluorescence fundus angiography and color fundus photography
◎: One of wide range, 4 fields (+center of macula), or 7 fields
●: Image centering the macula

n. If FA is conducted at screening, no need to repeat on Day 1.

o. To be performed at W52 or later (as a guide, at the visit next to a visit involving research drug administration).

p. ICGA will be performed only when deemed necessary by the investigator co-investigator.

q. Reported by patients via ePRO. If a patient has difficulty answering the questions by him/her-self due to visual impairment or other reasons, a family member or other person may read the questions aloud and enter the answers on the patient's behalf.

r. Patients will report their subjective symptoms such as visibility daily and record their life problems or motivation for treatment in arbitrary timings using an ePRO device.

* DME is regarded as active if CST is > 325 μm** and clinically significant*** IRF or SRF is observed. Determination of disease activity will be performed for the study eye only.

** > 325 μm with Spectralis SD-OCT and > 315 μm with Cirrus or Topcon SD-OCT (or other equivalent OCT)

*** The condition is considered clinically significant if it is deemed to be a cause of vision loss or other aggravation of the disease.

Appendix 2 Entry Items in eCRF

(1) Registration Form

| Survey Item | Collection Item |
|---|---|
| Patient Background | Patient identification number<br>Gender<br>Age at time of initial consent obtained<br>Ethnicity<br>Race |
| Planned start date of administration | Planned start date of administration |
| Study Eye | Study eye (right or left eye)<br>Whether or not an anti-VEGF drug was administered intravitreously to the study eye (if yes, date of last administration) |
| Informed Consent | Whether or not written consent is obtained<br>(If yes) Date of obtaining written consent |
| Inclusion and Exclusion Criteria | Inclusion criteria<br>Exclusion criteria |
| Medical history/complications | Medical history/complications (if yes, medical history or complication, name of disease) |
| History of diabetes mellitus | Diagnosis date of diabetes mellitus<br>Type of diabetes mellitus<br>Treatment for diabetes mellitus (if yes, type of anti-diabetic medication) |
| Surgical History | Surgical history (if yes, name of disease, name of operation, date of operation) |
| Surgical History (eye disease) | History of surgery (if yes, name of disease, eye(s) involved, name of procedure, date of surgery) |
| Pretreatment and Concomitant Medications | Drug name, route of administration, daily dose, start/end ate of administration or ongoing, reason for administration |
| Pre-treatment and Concomitant Medications (Ocular Diseases) | Drug name, eye of drug, route of administration, daily dosage, start/end date of administration or ongoing, reason for administration |
| Vital Signs | Systolic/diastolic blood pressure, pulse rate<br>Abnormality<br>Use of antihypertensive drugs |
| Height and Weight | Height, weight |
| Intraocular Pressure | Intraocular pressure (right and left eye) |
| Refraction Test | Spherical power, cylinder power, cylinder axis (right and left eye)<br>Abnormalities (right and left eye) |
| Visual Acuity Test | Visual acuity before DME (right and left eyes) Decimal acuity (right and left eyes)<br>Finger counting, hand movement, or light perception (for decimal acuity less than 0.02) |
| Slit-lamp Microscopy | Abnormalities (right and left eye) |
| Indirect | Abnormalities (right and left eye) |

| | |
|---|---|
| ophthalmoscopy | |
| SD-OCT | Model used, CST (right and left eye), presence/absence of pathologically significant IRF/SRF (right and left eye), presence/absence of active DME (study eye only) |
| Laboratory Test | HbA1c, Cr |

(2) Survey Form

| Survey Item | Collection Item |
|---|---|
| Visit | day of a visit to a hospital |
| Vital Signs | Systolic/diastolic blood pressure, pulse rate<br>Abnormality |
| Intraocular Pressure | Intraocular pressure before administration (right and left eye)<br>Intraocular pressure of the study eye (30 minutes after administration) |
| Visual Acuity Test | Decimal acuity test (right and left eyes)<br>Finger counting, hand movement, or light perception (for decimal acuity less than 0.02)<br>Finger counting (within 15 minutes after end of administration) |
| Slit-lamp Microscopy | Abnormalities (right and left eye) |
| Indirect Ophthalmoscopy | Abnormalities (right and left eye) |
| FA/ICGA | imaging date |
| Angiogenic glaucoma | Presence of neovascular glaucoma (study eye) |
| SD-OCT | Model used, CST (study eye), presence/absence of pathologically significant IRF/SRF (study eye), presence/absence of active DME activity (study eye) |
| Laboratory Test | HbA1c, Cr |
| Intravitreal administration of research drugs | Dose administered (if yes, date and dose)<br>(If dosage is changed, the reason for the change) |
| Concomitant Drugs | Presence/absence of concomitant medications (if yes, drug name, route of administration, daily dose, start date, treatment end date or ongoing, reason for administration) |
| Concomitant Therapy | Presence of concomitant therapy (if yes, name of therapy, date therapy started, date therapy ended or is continuing, reason for administration) |
| Adverse Event | Adverse event (if any, name of adverse event, date of onset, seriousness [if serious, reason for serious], severity, outcome and date of confirmation of outcome, relationship to study drug, action taken for study by an adverse event, lot number of last study drug administered before adverse event occurred) |
| Dosing Status | Confirmation Date<br>Continuation/Termination/Discontinuance (if discontinuance, date and reason for discontinuation) |
