# Supplementary material for: Optimization of individualized faricimab dosing for patients with diabetic macular edema: Protocol for the SWAN open-label, single-arm clinical trial
Source: PLoS One. 2024 Oct 10;19(10):e0311484. doi: 10.1371/journal.pone.0311484 (PMC11466402; doi:10.1371/journal.pone.0311484)
Supplement: Attachment — Submitted filename: Response to Reviewers.docx [file pone.0311484.s006.docx]

| **Editor** | **Author comments** | **Changes to manuscript** |
| --- | --- | --- |
| 3. Thank you for stating the following financial disclosure: "Supported by Chugai Pharmaceutical Co., Ltd. The sponsor participated in the study design; the writing of the report; and the decision to submit the paper for publication. Funding was provided by Chugai Pharmaceutical Co., Ltd for the study and third-party writing assistance, which was supplied by Trishan Gajanand, PhD, and Nicole Tom, PhD, of Envision Pharma Group."  Please state what role the funders took in the study. If the funders had no role, please state: ""The funders had no role in study design, data collection and analysis, decision to publish, or preparation of the manuscript.""  If this statement is not correct you must amend it as needed.  Please include this amended Role of Funder statement in your cover letter; we will change the online submission form on your behalf. | The funder participated in the study design, data collection and analysis, the decision to publish, and in the preparation of the manuscript – this statement has been included in the cover letter as requested. | No change to the manuscript – the role of the funder has been amended in the cover letter. |
| 4. Thank you for stating the following in the Competing Interests section: "Takao Hirano: Speaker Fees: Bayer, Canon, Chugai Pharmaceutical Co., Ltd., Kowa, Novartis Pharma KK, Santen Pharmaceutical Co., Senju, ZEISS Toshinori Murata: Speaker Fees: Bayer, Novartis, Santen, Zeiss Meditec: Consultant: Boehringer Ingelheim, Chugai Pharmaceutical Co., Ltd., Hoya, Kowa, Wakamoto Shintaro Nakao: Consultant: Alcon, Boehringer Ingelheim, Novartis, Riverfield; Speaker Fees: Bayer, Boehringer Ingelheim, Chugai Pharmaceutical Co., Ltd., Hoya, Kowa, Machida, Mitsubishi Tanabe, Novartis, Novo Nordisk, Otsuka, Santen, Senju, Wakamoto Masahiko Shimura: Consultant: Bayer, Boehringer Ingelheim, Chugai, HOYA, Nikki HD, Roche, Wakamoto; Lecture Fees: Bayer, Chugai, Kowa, Novartis, Otsuka, Senju Miho Nozaki: Speaker Fees: Bayer, Canon, Chugai Pharmaceutical Co., Ltd., Kowa, Nikon, Novartis Pharma KK, Santen, Senju, Sumitomo Pharma Co., Ltd., Topcon Medical, Wakamoto Kiyoshi Suzuma: Speaker Fees: AMO, Alcon, Bayer, Chugai Pharmaceutical Co., Ltd., HOYA, Kowa, Novartis, Senju Consultant: Senju, Chugai Pharmaceutical Co., Ltd., Boehringer Ingelheim Taiji Nagaoka: Consultant: Boehringer Ingelheim, Novartis, TES Holdings; Speaker Fees: Bayer, Chugai Pharmaceutical Co., Ltd., Hoya, Kowa, Mitsubishi Tanabe, Novartis, Santen, Senju, Wakamoto; Research Fees: Daicel, Kowa, LTT, Santen Masahiko Sugimoto: Research Fees: Alcon Japan, Bayer, Chugai Pharmaceutical Co., Ltd., Novartis; Speaker Fees: Alcon Japan, Bayer, Kowa, Novartis, Senju, Wakamoto Yoshihiro Takamura: Lecture Fees: Chugai Pharmaceutical Co., Ltd.  Tomoaki Murakami: Speaker Fees: Bayer, Canon, Chugai Pharmaceutical Co., Ltd., Johnson & Johnson, Kowa, Novartis Pharma KK, Santen; Consultant: Boehringer Ingelheim Keisuke Iwasaki: Employee: Chugai Pharmaceutical Co., Ltd.  Jun Tsujimura: Employee: Chugai Pharmaceutical Co., Ltd. Shigeo Yoshida: Consultant: Chugai Pharmaceutical Co., Ltd., Novartis; Speaker Fees: Bayer, Chugai Pharmaceutical Co., Ltd., Novartis, Senju; Research Fees: Kowa, Otsuka, Senju  "  Please confirm that this does not alter your adherence to all PLOS ONE policies on sharing data and materials, by including the following statement: ""This does not alter our adherence to  PLOS ONE policies on sharing data and materials.” (as detailed online in our guide for authors [http://journals.plos.org/plosone/s/competing-interests](https://url.us.m.mimecastprotect.com/s/dgKTC9rPWviM7pvBSZbyo4?domain=journals.plos.org)).  If there are restrictions on sharing of data and/or materials, please state these. Please note that we cannot proceed with consideration of your article until this information has been declared.  Please include your updated Competing Interests statement in your cover letter; we will change the online submission form on your behalf. | We confirm that the author competing interests do not alter our adherence to PLOS ONE policies on sharing data and materials – this statement has been included in the cover letter as requested. In addition, we have added the following data sharing statement to the submission form.  The datasets generated during and/or analysed during the current study are available from the corresponding author on reasonable request. | None |
| 5. Please provide a complete Data Availability Statement in the submission form, ensuring you include all necessary access information or a reason for why you are unable to make your data freely accessible. If your research concerns only data provided within your submission, please write "All data are in the manuscript and/or supporting information files" as your Data Availability Statement. | We have added the following data sharing statement to the submission form.  The datasets generated during and/or analysed during the current study are available from the corresponding author on reasonable request. | None |
| 6. Please include captions for your Supporting Information files at the end of your manuscript, and update any in-text citations to match accordingly. Please see our Supporting Information guidelines for more information: http://journals.plos.org/plosone/s/supporting-information. | Captions for the supporting information files have now been included at the end of the manuscript. | The following has been added to the end of the manuscript:  **“Supporting Information**  **Table S1. SWAN trial eligibility criteria**  **Table S2. SWAN trial prespecified endpoints**  **Figure S1: YOSEMITE and RHINE Trial Design”** |
| 7. Please review your reference list to ensure that it is complete and correct. If you have cited papers that have been retracted, please include the rationale for doing so in the manuscript text, or remove these references and replace them with relevant current references. Any changes to the reference list should be mentioned in the rebuttal letter that accompanies your revised manuscript. If you need to cite a retracted article, indicate the article’s retracted status in the References list and also include a citation and full reference for the retraction notice. | We have updated reference 6 (Wong TY et al. *Ophthalmology*. 2024) as this was previously online only but has now been published.  We have updated reference 8 (Shimura M et al. *Jpn J Ophthalmol*. 2024) as this was previously under review but is now published online. | References 6 and 8 have been updated as follows:  6. Wong TY, Haskova Z, Asik K, Baumal CR, Csaky KG, Eter N, et al. Faricimab Treat-and-Extend for Diabetic Macular Edema: Two-Year Results from the Randomized Phase 3 YOSEMITE and RHINE Trials. Ophthalmology. 2024;131(6):708-23. Epub 2024/01/02. doi: 10.1016/j.ophtha.2023.12.026.  8. Shimura M, Oh H, Ueda T, Kitano S, Mitamura Y, Sato J, et al. Efficacy, durability, and safety of faricimab with extended dosing up to every 16 weeks in diabetic macular edema: 2-year results from the Japan subgroup of the phase 3 YOSEMITE trial. Jpn J Ophthalmol. 2024. Epub 2024/07/31. doi: 10.1007/s10384-024-01078-y. |
| **Reviewer 1** | **Author comments** | **Changes to manuscript** |
| This manuscript is a introduction of SWAN trial, a 2-year, open-label, single-arm, interventional, multicenter trial enrolling adults with center-involving DME treated with faricimab 0.6mg.  Improvement of vision outcomes and reduction of treatment burden are warranted for anti-VEGF therapy and faricimab is expected to resolve such unmet needs.  The SWAN trial is excellently organized prospective study. I am looking forward to the authors’ continue success.  I think that the manuscript is suitable for the publication in the PLOS ONE. | We thank the Reviewer for these comments. | None. |
| **Reviewer 2** | **Author comments** | **Changes to manuscript** |
| From the statistical viewpoint, it is a curiosity that the authors did not consider possibility of using an adaptive design for a single arm trial. It is helpful such a discussion be included in the paper. The adaptive design has the potential of requiring fewer patients to be enrolled and less time to conclude whether the proposed treatment is effective or not. For example, a most common trial of this kind is the celebrated Simon Two-Stage design for a Phase 2 trials discussed and implemented in clinical trials with many variations thereof. It has two stages and assumes the primary endpoint is binary (responders or not) and depending on the observed proportion of responders in stage 1, the trial may or may not move to the second stage to continue to assess the efficacy of the treatment. Here a null hypothesized success rate is postulated along with an alternative hypothesis that postulates a higher rate of success. The strategy is that if the null hypothesis is not met, the treatment is not effective and the trial terminates. If it is not rejected the trial proceeds to the second stage. In each stage, the number of patients and number of responders have to be determined after the user-selected type 1 and 2 errors for testing the hypotheses are specified. The design problem is then to determine the number of patients and the number of responders required in Stage 1, and if the trial proceeds to Stage 2, the additional number of patients needed and the additional number of responders needed to conclude efficacy of the treatment. | We thank the reviewer for these comments and for this suggestion. Unfortunately, we will not be able to change the study design because the SWAN trial started in August 2023.  We did not use an adaptive study design for this study as the purpose of the trial is to evaluate the efficacy and durability of faricimab during the maintenance period under real-world clinical conditions for DME. Specifically, accumulating long-term data from all cases itself is of importance in this study, and we are not looking to introduce criteria for discontinuation due to inefficacy. In addition, we aim to assess the impact of adjusting the dosing interval on efficacy. If we included discontinuation criteria for inefficacy, there is a possibility that these aspects may not be adequately evaluated. For these reasons, the study was designed as described. | None. |
| It was stated in the abstract and elsewhere that “The primary endpoint is change in best-corrected visual acuity (BCVA) from baseline at 1 year (averaged over weeks 52, 56, and 60)” This seems unclear to me what is meant by the averaging referred to in the parentheses. Please clarify. | The visit when the study drug is administered varies by patient, with some patients receiving faricimab at week 48 and others at week 52. Therefore, to minimize the effect of differences in time from last treatment, and to account for best-corrected visual acuity (BCVA) variability over time, the primary endpoint was averaged over weeks 52, 56, and 60. This approach was also used in the phase 3 YOSEMITE and RHINE trials (Wykoff CC et al. *Lancet*. 2022. 399(10326):741-755.   doi: 10.1016/S0140-6736(22)00018-6.) | None. |
| In the abstract, not clear secondary/exploratory endpoints mean. Are you treating secondary and exploratory endpoints equivalent? If not, make clear what are secondary endpoints and what are exploratory endpoints. The result sections can use a rewrite to make clearer each of the variables involved and the threshold for success – the sentence right now is hard to understand because it is long and somewhat convoluted. | We have removed the term “exploratory” from the Results section of the Abstract for clarity. In addition, the text “*proportion of patients who achieve Q24W dosing*” has been removed. | The second sentence of the Results section of the Abstract has been adjusted to the following:  “*Key secondary endpoints include: change from baseline in BCVA, CST, and National Eye Institute Visual Function Questionnaire scores over time; proportion of patients with BCVA (decimal visual acuity) ≥0.5, ≥0.7, ≥1.0, or ≤0.1; proportion of patients with absence of DME, and IRF and/or SRF over time*.” |
| The description of the proposed trial and the two previous trials (YOSEMITE (NCT03622580) and RHINE (NCT03622593)) should be contrasted in a diagram or schematic form to facilitate understanding of the treatment regimen involved. Right now, they are wordy going from lines 69 to 90, which can cloud comprehension. | The study design schematic for the YOSEMITE and RHINE trials has been added to the manuscript as a supplementary figure.  We have also edited the text describing the SWAN and YOSEMITE/RHINE trials in the Introduction for clarity. | We have removed the following from the second paragraph of the Introduction:  “*In addition, more faricimab-treated patients than aflibercept-treated patients achieved intraretinal fluid (IRF) absence and* *central subfield thickness (CST) <325 µm through year 2 [6].*”  We have adjusted the final sentence of the second paragraph of the Introduction to the following:  “*Furthermore, a subsequent YOSEMITE and RHINE post hoc analysis found that 56% of patients who achieved Q16W faricimab dosing met the criteria for potential extension to every 20 weeks (Q20W) dosing [9].*”  We have added the YOSEMITE/RHINE trial design figure as supplementary with the following title:  “*Figure S1: YOSEMITE and RHINE Trial Design*” |
| Statistical analyses:  In the sample size calculation, the study is powered for detecting a postulated difference. Can the author discussed whether the postulated difference of interest is a meaningful outcome from the clinical perspective since a significant difference may not correspond to a clinically meaningful difference. | A clinically meaningful difference is 5 letters on Early Treatment Diabetic Retinopathy Study, which is an improvement of approximately 0.1 when converted to logMAR. The SWAN study was designed to assume an improvement of 0.22 when converted to logMAR, and the sample size was determined with consideration for this clinically meaningful difference. | None. |
| A 35% dropout rate is anticipated in the first year. Please justify where this number is coming from and whether the proposed analyses account for possible values other than a 35% dropout rate. | A dropout rate of 35% is estimated for the first year of the SWAN trial due to the burden of treatment costs and visits.  In the YOSEMITE/RHINE trials, the dropout rate over one year was approximately 10%; however, in the current study, since patients will be responsible for the cost of faricimab, and because of the burden of hospital visits, a dropout rate of 35% is anticipated during the first year. The target number of patients was set at 70. | None. |
| There are several secondary variables to be evaluated, such as determining the proportions of patients who meet predefined thresholds for BCVA (i.e., decimal visual acuity ≥0.5, ≥0.7, ≥1.0, or ≤0.1) over time, in addition to many others. Is there going to be a single composite measure that describes whether the patients did well or not, based on the secondary variables? | No single composite measure based on the secondary variables will be used in this study. Each binary endpoint was designed to evaluate the improvement and maintenance of visual acuity using multiple thresholds for BCVA. | None. |
| Remaining comments pertain to the last paragraph prior to “Trial status” on page 25, where the authors stated that “BCVA analyses will follow the mixed effect model for repeated measures, which will include visit (categorical variable) and baseline BCVA (continuous variable) as fixed effects and will assume an unstructured covariance structure for modeling intrasubject error. For missing data, missing-at-random will be assumed.”a. Why is the variable 'visit' is treated as a categorical variable and not as a continuous variable in real time? In the trial, patients are supposed to visit at designated time points, and they rarely do so in practice. What happens if patients show up at different time points? What is the threshold before the reading is considered invalid because the timing is unacceptably different from that specified in the protocol? For example, patients are required to visit a clinic for testing at least every 2 months – what happens if patient show up ½ month late? Is this a valid data point? Further, treating the variable visit as a categorical variable has implicit constraints imposed on the interpretation of the results. | We thank the Reviewer for their questions regarding the mixed effect model for repeated measures.  In the YOSEMITE/RHINE trials, the measurement taken closest to the reference visit date at the relevant visit, including measurements taken for unscheduled visits, was adopted for each visit and was included in covariates for MMRM analyses as categorical variables.  The same approach will be used in the current study. By using this approach, it will be possible to estimate differences for each visit (as categorical). | None. |
| b. Please make clearer what are the random components in the mixed effects model alluded to? | Of interest to the current study is the average effect on the treatment arm. In mixed model for repeated measures analysis, as described by Mollinckrodt et al., (2008), random effects are considered in the variance-covariance structure and modelled, but are not explicitly specified in the analysis. For this reason, random effects are not specified in the mixed model for repeated measures analysis in the current study.  Mallinckrodt, et al (2008). *Ther Innov Regul Sci* 42, 303–319 | None. |
| c. Are the authors going to fit other covariance structures and select the best fitting model for prediction purposes? | The variance-covariance structure will be unstructured by default. Only if the model does not converge, due to a small sample size or any other relevant reason, will we attempt to converge the model by re-specifying other pre-specified variance-covariance structures (such as compound symmetry).  Please note that the purpose of this study was to estimate the average treatment effect in the study arm; this was not a prediction analysis. | None. |
| d. To ensure reproducibility, please mention what statistical package or software is going to be used to analyze the data. | We will use SAS 9.4 to analyze the data. This detail has been added to the Methods of the manuscript. | We have added the following as the first sentence of paragraph 2 of the Statistical approaches section of the Methods:  “*Data will be analyzed using SAS 9.4 (SAS Institute Inc., Cary, NC).*” |
| e. Missing data are assumed to be missing-at-random. It is helpful to elaborate how the authors are going to check whether such an assumption is valid – in particular, are statistical diagnostic tools going to be employed to ascertain whether such an assumption seems valid, and if so how? | If validation of missing-at-random data is required, we will complete sensitivity analyses using the last observation carried forward method for imputing missing outcome data to confirm that results are not substantially different from the primary analysis. | None. |
